# Morphology-Dependent Interaction of Silica Nanoparticles with Intestinal Cells: Connecting Shape to Barrier Function

**DOI:** 10.1021/acs.nanolett.3c00835

**Published:** 2023-07-11

**Authors:** Claudia Iriarte-Mesa, Maximilian Jobst, Janice Bergen, Endre Kiss, Ryong Ryoo, Jeong-Chul Kim, Francesco Crudo, Doris Marko, Freddy Kleitz, Giorgia Del Favero

**Affiliations:** † Department of Inorganic Chemistry−Functional Materials, Faculty of Chemistry, University of Vienna, Währinger Str. 42, 1090 Vienna, Austria; ‡ Vienna Doctoral School in Chemistry (DoSChem), University of Vienna, Währinger Str. 42, 1090 Vienna, Austria; ∥ Core Facility Multimodal Imaging, Faculty of Chemistry, University of Vienna, Währinger Str. 38-40, 1090 Vienna, Austria; ⊥ Department of Food Chemistry and Toxicology, Faculty of Chemistry, University of Vienna, Währinger Str. 38-40, 1090 Vienna, Austria; # Department of Energy Engineering, Korea Institute of Energy Technology (KENTECH), 21 KENTECH-gil, Naju 58330, Republic of Korea; ∇ Center for Nanomaterials and Chemical Reactions, Institute for Basic Science (IBS), Daejeon 34141, Republic of Korea

**Keywords:** silica nanoparticles, intestinal cells, membrane permeation, morphology-dependent interaction, mucus barrier

## Abstract

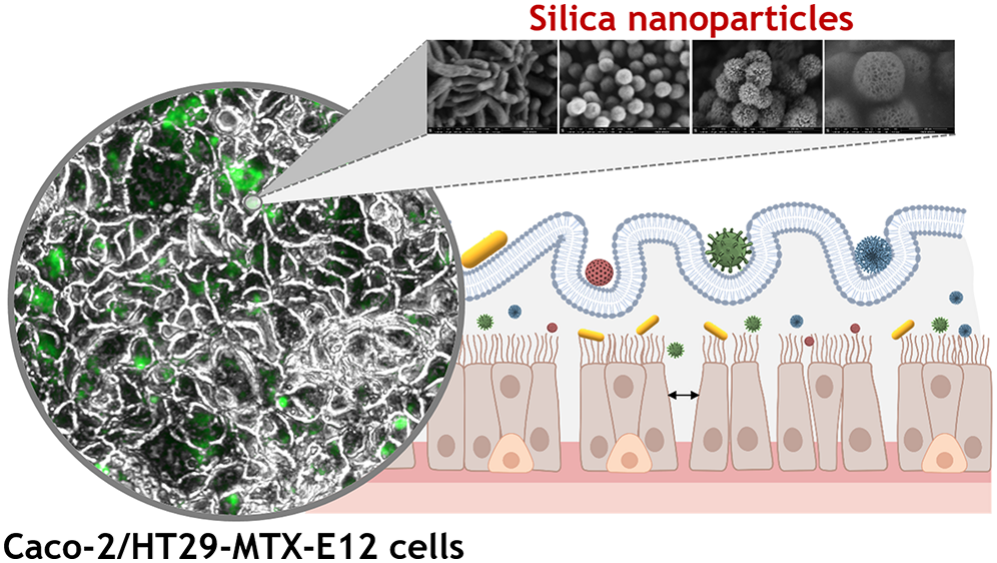

The intestinal compartment ensures nutrient absorption and barrier function against pathogens. Despite decades of research on the complexity of the gut, the adaptive potential to physical cues, such as those derived from interaction with particles of different shapes, remains less understood. Taking advantage of the technological versatility of silica nanoparticles, spherical, rod-shaped, and virus-like materials were synthesized. Morphology-dependent interactions were studied on differentiated Caco-2/HT29-MTX-E12 cells. Contributions of shape, aspect ratio, surface roughness, and size were evaluated considering the influence of the mucus layer and intracellular uptake pathways. Small particle size and surface roughness favored the highest penetration through the mucus but limited interaction with the cell monolayer and efficient internalization. Particles of a larger aspect ratio (rod-shaped) seemed to privilege paracellular permeation and increased cell–cell distances, albeit without hampering barrier integrity. Inhibition of clathrin-mediated endocytosis and chemical modulation of cell junctions effectively tuned these responses, confirming morphology-specific interactions elicited by bioinspired silica nanomaterials.

Intestinal cells constantly face a wide variety of chemical challenges; this includes exposure to food constituents and contaminants, drugs, and microbial communities and their metabolites.^1−3^ Additionally, physical cues originating from the peristaltic movements support the chyme transit and exert a trophic function on the tissue.^4,5^ In relation to these complex chemical and physical stimuli, the membrane of intestinal cells is exposed to the particulate matter of the food chyme, as well as to the microbiome populating the luminal cavity. This leads to the fundamental question of how cells can potentially adapt their morphology and barrier function when exposed to particles of different shapes, and if this aspect can contribute to the effects triggered by biochemical stimuli.

Mesoporous silica nanoparticles (MSNs) have proven to be excellent drug carriers over the last years. Their high biocompatibility, stability, rigid framework, high surface-to-volume ratio, well-defined pore structure, and tunable surface chemistry have catalyzed the interest in silica-based formulations for oral drug delivery.^6−8^ This in turn has encouraged the development of novel methodologies for the synthesis of silica materials capable of overcoming both the gastric barrier and physicochemical challenges in the gut, including the mucus layer, cell tight junctions, and digestive enzymes.^7,9^ The influence of physicochemical properties on the particle biodistribution, excretion, and toxicity has been studied.^10^ In the design of nanocarriers, special emphasis has been placed on the contribution of charge, morphology, and particle size, all of which modulate the interaction with the cell membrane, cellular uptake, and intestinal permeation.^11−13^ Additionally, the synthesis of particles with controlled size can support penetration through the mucus layer.^14,15^ However, the biological characterization of these materials returns quite a complex activity profile, and much remains to be explored of the mechanisms behind the reported results. For example, it has been described that particles smaller than 50 nm with highly negative surface charges are able to pass through the intestinal membrane^12,16,17^ but have in turn low stability in blood circulation. In contrast, medium-sized particles (i.e., 100–150 nm) are not as efficiently taken up by cells, but they have a longer permanence in the body fluids.^18^ Furthermore, virus-like particles of comparable sizes have displayed unique internalization pathways and extended blood circulation time due to the biomimetic morphology and surface roughness.^13,19,20^ Additionally, compared to spheres of the same chemistry, rod-shaped particles displayed enhanced transport and trafficking capabilities in the interaction with the intestinal mucus layer.^11,14,21,22^ Indeed, smooth spherical particles can get trapped and removed by mucus, thereby limiting the effectiveness of such drug delivery systems.^23^ Once the mucus barrier has been crossed, nanomaterials either interact with the cell membranes to initiate intracellular uptake, or privilege the paracellular route by loosening the cell junctions.^24,25^ Several endocytic pathways have been described for the internalization of MSNs, including nonspecific cellular uptake mechanisms in which the physicochemical properties of the nanoparticles display a fundamental role.^26−28^ Even though much remains to be understood about shape-dependent responses, it is clear that the generation of nanomaterials with tunable bioinspired morphologies holds an incredible potential. This approach promises to offer complementary tools to functionalization and surface chemistry in order to achieve unprecedented biological applications. In addition to the obvious correlation to pathophysiology,^29^ in-depth understanding of the shape–activity profile could greatly support the design of materials and carriers with differential cell uptake potentials and a controllable ability to interact with the intestinal barrier. Taking this as starting point, bioinspired materials were generated. Contributions of different sizes and particle morphologies were evaluated, which included spherical (i.e., dendritic mesoporous silica nanoparticles, DMSNs), virus-like (**VlNPs**), and rod-shaped (**NrNPs**) nanoparticles. The structure–activity relationship was designed considering crucial aspects of the preservation of intestinal barrier integrity, such as the intra-pericellular localization of the particles and the cell–cell distance. The silica nanoparticles were modeled according to established protocols,^14,16,19,30^ and the synthesis conditions were adjusted to finely control size, morphology, and surface roughness in order to generate well-calibrated particles. Four different materials were obtained: DMSNs with a spherical diameter of 90 nm (**D**_**90**_), DMSNs with a spherical diameter of 130 nm (**D**_**130**_), **VlNPs** (virus-like, 130 nm), and **NrNPs** (rod-like, 160 nm x 35 nm), exhibiting hydrodynamic diameters of 161 (±2), 174 (±3), 166 (±1), and 216 (±3) nm, respectively (Figure S1a). Representative TEM and SEM images confirmed the achievement of controlled shape and size (Figure 1). The detailed characterization of the structure, surface charge, and porosity of the silica materials can be found in Figure S2 and Table S1.

**Figure 1:**
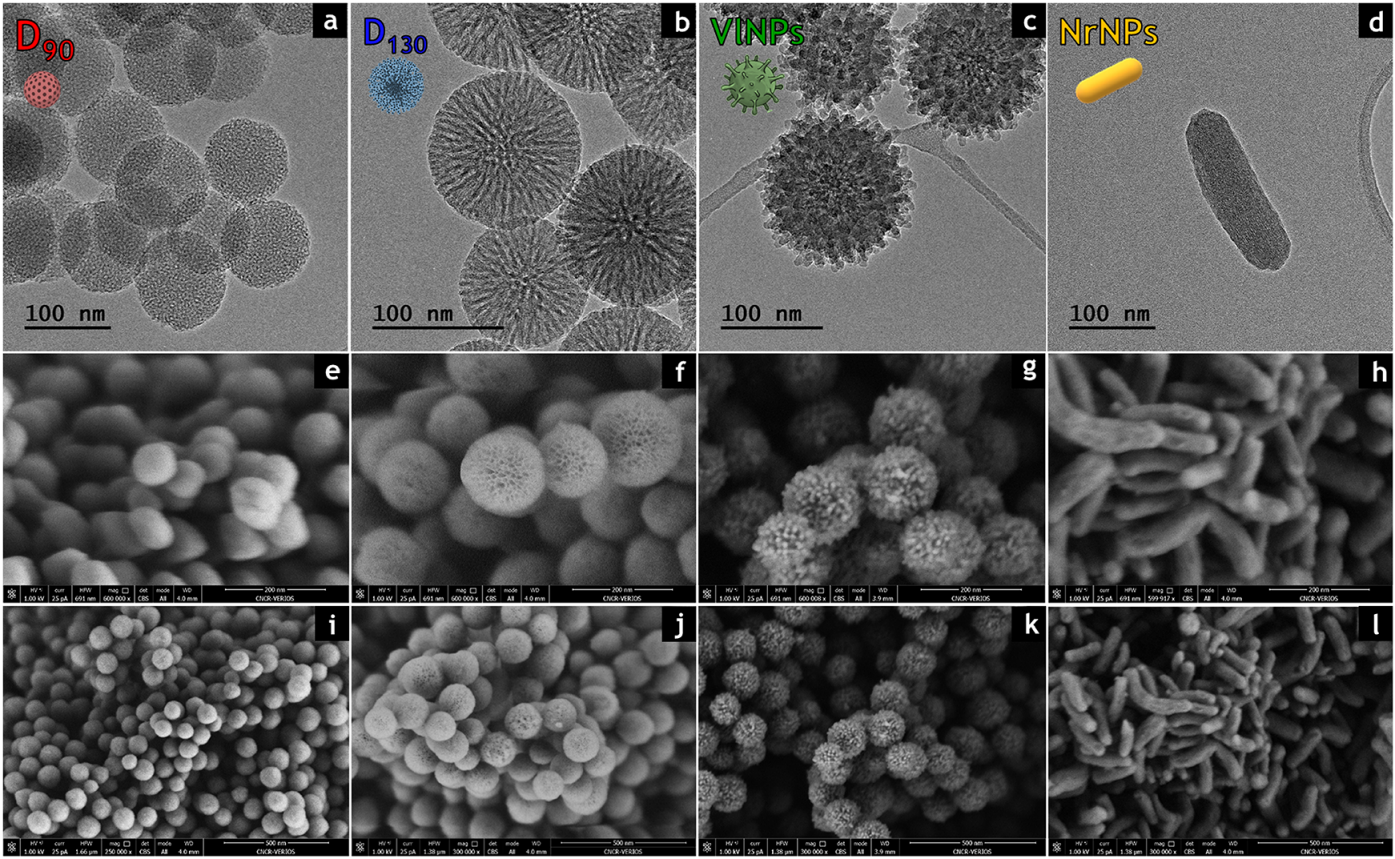
Representative transmission electron microscopy (TEM) images of (a) **D**_**90**_, (b) **D**_**130**_, (c) **VlNPs**, and (d) **NrNPs**. Scale bars represent 100 nm. Scanning electron microscopy (SEM) images of (e and i) **D**_**90**_, (f and j) **D**_**130**_, (g and k) **VlNPs**, and (h and l) **NrNPs**. Scale bars represent (e–h) 200 and (i–l) 500 nm.

To deepen on the capacity of tailored silica particles to diffuse through the mucus layer and to interact with intestinal cells, we took advantage of a differentiated coculture of Caco-2/HT29-MTX-E12 cells. This model has been previously used for studying the pathophysiological behavior of intestinal cells and the interaction with food constituents and contaminants.^31−33^ After 7 days of differentiation, cells form a tight epithelial monolayer that resembles salient features of the intestinal compartment in vivo. This includes the formation of villi-like structures, expression of tight junctions, more physiological glucose transport, and chloride and mucus secretion.^31,32^ The interaction of FITC-labeled nanomaterials, i.e., **D**_**90**_, **D**_**130**_, **VlNPs**, and **NrNPs**, with differentiated cocultured Caco-2/HT29-MTX-E12 was observed via live cell imaging (6 h treatment, 37 °C). To explore the role of clathrin-mediated endocytosis, the coated-pits inhibitor Pitstop 2 was included in the experimental layout (+ Mucus, ± Pitstop 2) (Figure S3). Pitstop 2 has been extensively used for evaluating the intestinal uptake of nanoparticles, since this inhibitor affects most of the endocytic pathways by interfering with binding of proteins to the N-terminal domain of clathrin.^26,34,35^ Furthermore, to isolate the contribution of the mucus layer, the experiments were carried out after the removal of the mucus with acetylcystein (− Mucus, ± Pitstop 2), according to a previously reported procedure.^36^ In order to quantify particles interacting with the intestinal cells after 6 h of treatment, the loose ones were removed by washing and the residual fluorescence was measured. To limit quantification artifacts or bias due to the selection of optical fields, experiments were performed with two independent imaging systems. First, high-magnification (63×) 3D reconstructions were obtained with confocal microscopy. Additionally, phase contrast was combined with fluorescence imaging at lower magnifications (10×) for the appreciation of larger fields of view. In several experimental conditions, the particles’ fluorescence detected in absence of mucus (Figure 2, a and b) returned higher intensities in comparison to data acquired in the presence of mucus (Figure S3), supporting the view that the mucus could effectively tune the penetration of the materials as well as reduce/slow down the interaction with the cell surface. For both quantification strategies, 63× magnification (Figure 2, c and d) and 10× magnification (Figure 2, e and f), the particles’ signal in control conditions (i.e., – Pitstop 2) was higher in comparison to the incubation with the inhibitor, which indicates the involvement of clathrin-mediated endocytosis in the cell–particle interaction (Figure 2, c–f). Particularly, in absence of mucus, the preincubation with Pitstop 2 further hampered the intensity of the detected signals. This effect was more evident for **VlNPs** and **NrNPs**, where a clear drop in the fluorescence intensities could be observed (Figure 2, d and f).

**Figure 2:**
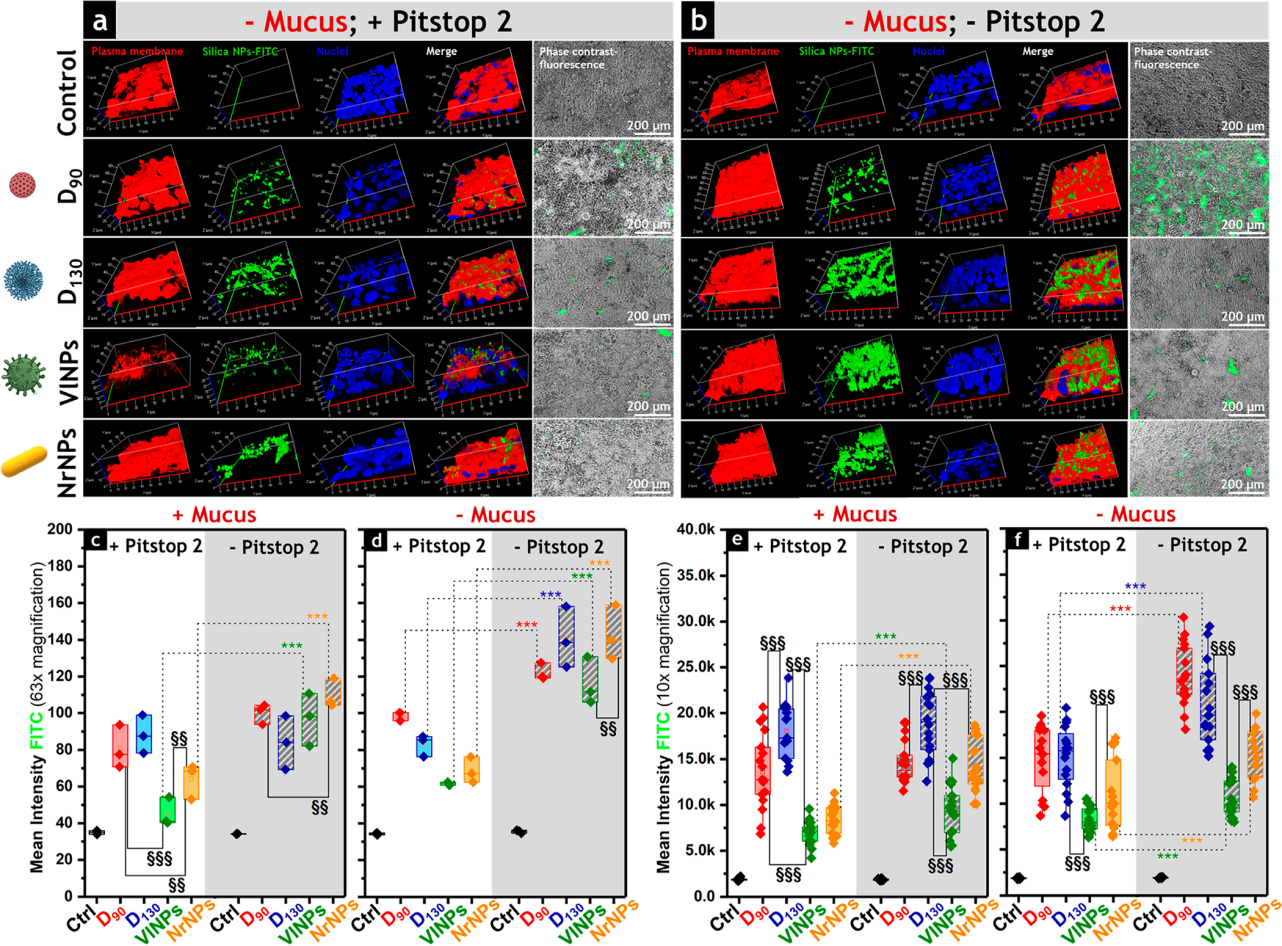
Representative live cell fluorescence images of the interaction of FITC-labeled silica nanoparticles with Caco-2/HT29-MTX-E12 after 6 h of incubation in the absence of mucus and previous treatment (a) with or (b) without Pitstop 2. In the 3D reconstructions (63× magnification), the scale bar segmentation is 10 μm, the plasma membrane is presented in red, FITC-labeled materials are presented in green, and cell nuclei are presented in blue. In the phase contrast images (10× magnification) scale bars represent 200 μm. Quantification of the mean fluorescence intensity of FITC from (c and d) confocal microscopy (*n* = 3 optical fields) and (e and f) phase-contrast fluorescence (*n* = 18 optical fields) in (c and e) the presence and (d and f) the absence of mucus. Statistically significant differences according to one-way ANOVA and Fisher Test when treatments compare the same particle type (*) or different ones (§) are as follows: */§ (*p* < 0.5), **/§§ (*p* < 0.01), or ***/§§§ (*p* < 0.001). All data were obtained from three independent cell preparations.

For the **D**_**130**_-treated cells, the inhibitor effect was only significant in the absence of mucus, which could likely be attributed to a limited transport of medium-sized spherical particles through this barrier.^15^ Overall, the results underpinned that the particle–cell interaction depends on particle morphology and is highly affected by the presence of mucus.

To verify whether the treatment with Pitstop 2 could potentially account for a change of cell elasticity and eventually justify the altered efficiency of interaction with nanoparticles, atomic force microscopy (AFM) experiments were performed. Measurements of the Young’s modulus of cells after mucus removal (Figure S4) revealed an increase of cell stiffness after treatment with Pitstop 2 compared to the negative control (− Mucus, – Pitstop 2). This supports the interpretation of a possible lower compliance of the Caco-2/HT29-MTX-E12 cells to take up the nanoparticles via an endocytosis-mediated mechanism. Furthermore, cell surface profiling in the presence of mucus obtained via tapping mode suggested that the process of mucus removal was well tolerated by the monolayer. This supported the notion that the differences in the cell–particle interaction could be more likely attributed to the presence or absence of the mucus barrier rather than to unspecific cytotoxicity or a loss of cell morphology.

In order to limit the possibility that the observed effects could be due to technical artifacts, i.e., uneven distribution of the particle suspension on the complex topology of the cell monolayer, additional experiments were performed focusing on the particle–cell layer interaction with (Figure 3a) and without mucus (Figure S5). Cells were imaged at the same coordinates immediately after the application of the particles (*t*_0_) but also after 6 h of treatment (Figure S6). For the latter, two images were acquired, one maintaining exactly the same focus as that for *t*_0_ and the second one refocusing on the particle layer (*t*_6_). Being that the image focus is dependent on the position of the sample, the difference between the two acquisitions (*t*_0_ – *t*_6_) was used to calculate the penetration of the materials toward the cell monolayer (Figure 3, b and c). As visible in Figure 3b, broader distribution of the data was observed when experiments were performed in the presence of mucus, most likely as a result of particle “trapping” within the respective layer. **NrNPs**, **D**_**90**_, and **VlNPs** were the materials that more easily diffused through the mucus barrier, with 45, 36, and 32 μm focus adjustments, respectively (Figure 3b, – Pitstop 2). In the absence of mucus, particles had reduced movement capacity, resulting on average in lower penetration along the *z*-axis (Figure 3c). The presence of the inhibitor Pitstop 2 returned even smaller adjustment between the focus fields, which is compatible with reduced particle–cell interactions (Figure 3c).

**Figure 3:**
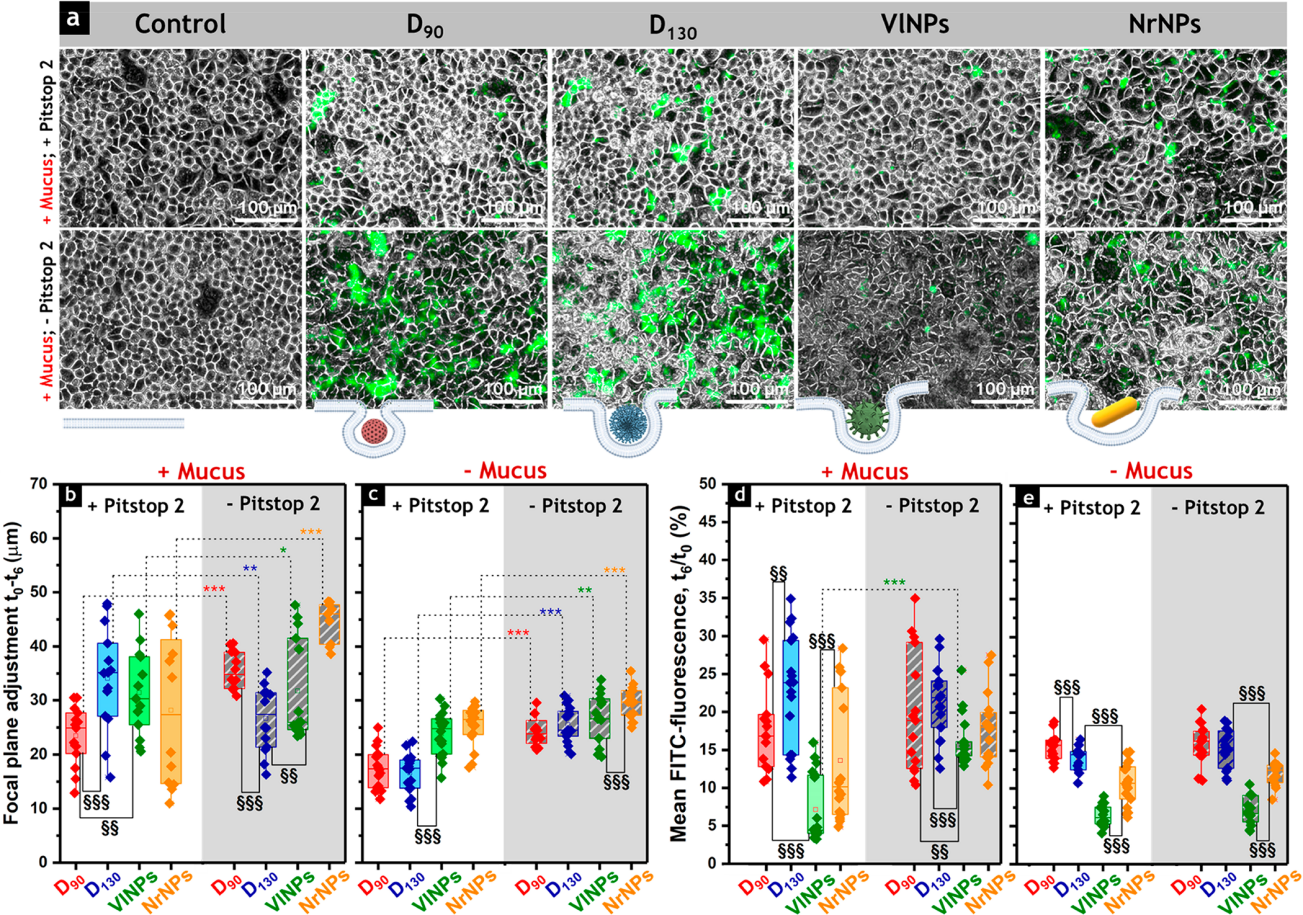
(a) Appearance of Caco-2/HT29-MTX-E12 after treatment with FITC-labeled particles (20× magnification). Scale bars represent 100 μm. (b and c) Quantification of the focal plane adjustment obtained from the difference between the optical parameters set immediately after treatment with the FITC-labeled particles (*t*_0_) and after 6 h of incubation (*t*_6_) for cells with or without the mucus layer. (d and e) Quantification of the residual FITC fluorescence signal due to particle–cell interaction with or without the mucus layer (%). At least 18 paired images were analyzed before and after focus adjustment (*n* = 18). Statistically significant differences according to one-way ANOVA and Fisher Test when treatments compare the same particle type (*) or different ones (§) are as follows: */§ (*p* < 0.5), **/§§ (*p* < 0.01), or ***/§§§ (*p* < 0.001). All data were obtained from three independent cell preparations.

In order to further characterize the fluorescence signal variation within 6 h of incubation in relation to the initial conditions of the samples, images acquired at the beginning (*t*_0_) and at the end of the experiment (*t*_6_) were compared (Figure 3, d and e). Since the data pattern largely aligned with the quantification values previously obtained (Figure 2, c–f), it can be assumed that differences among the treatments were more likely related to the behavior of particles with tuned morphology and size. Additionally, the *t*_0_ images confirmed that the nanoparticles were homogeneously distributed at the beginning of the workflow (Figure S6), in agreement with the measurements of colloidal stability over time followed by DLS (Figure S1b).

Reading across, **NrNPs** displayed the highest efficiency of penetration through the mucus layer (Figure 3, b and c). However, looking at the cell monolayer, rod-shaped particles returned rather low fluorescence intensities (%), possibly indicating reduced interactions (Figure 3, d and e). Of note, the hydrodynamic diameter of **NrNPs** increased from 216 (±3) to 330 (±10) nm when the particles were dispersed in cell culture medium instead of nanopure water (Figure S7). This significant increase was attributed to the lack of colloidal stability in the biological medium, as reflected by polydispersity indexes (PDI) above 0.3 (Figure S7). Aggregation and lower colloidal stability would be in agreement with limited interaction of **NrNPs** with the cell surface.

To further expand on the influence of size on the interaction of silica nanoparticles with the intestinal cells, smaller spheres were also generated (spherical diameter of 35 nm, **D**_**35**_). **D**_**35**_ showed a hydrodynamic diameter of 55 (±1) nm (Figure S1). The synthesis of **D**_**35**_ was optimized (structure and porosity of **D**_**35**_ can be found in Figures S2 and Table S1) to ensure not only a smaller spherical diameter than **D**_**90**_ and **D**_**130**_ but also dimensions comparable to the thickness of the **NrNPs** (Figure S8a). The interaction of **D**_**35**_ with the Caco-2/HT29-MTX-E12 monolayer was evaluated by live cell imaging (phase contrast and fluorescence; Figure S8b) following the same experimental procedure used for the other materials (± Mucus, ± Pitstop 2). After 6 h of incubation, **D**_**35**_ exhibited the highest focal plane adjustment (42 μm, *t*_0_ – *t*_6_) with respect to the other spherical particles in control conditions (i.e., + Mucus, – Pitstop 2; Figure 3b), following the order of hydrodynamic diameter (Figure S8c). This behavior confirmed that the silica diffusion capacity through the mucus layer is limited when size increases. In addition, the mean FITC-fluorescence intensities obtained after 6 h of incubation (Figure S8d) were lower in comparison to those obtained for **D**_**90**_ and **D**_**130**_ (Figure 2, e and f). This might suggest that the smaller the size, the less efficient interaction of spherical particles with the cell layer is. These experiments additionally provided a strong indication that the enhanced penetration efficiency of rod-like particles through the mucus layer may be associated with their smallest diameter perpendicular to the longest axis, since similar performance was observed for both **D**_**35**_ and **NrNPs** in all the conditions tested (± Mucus, ± Pitstop). The higher diffusivity and superior transport and trafficking capability of **NrNPs** in mucus, leading to deeper penetration through this barrier, could also be attributed to the rotational dynamics of the rod-shaped particles, as previously reported.^14^

Overall, imaging experiments suggested that despite the interaction of particles with the cell monolayer, the materials were scarcely observed inside the cells and rather distributed on the outer surface. Therefore, the possibility of paracellular permeation was explored. Based on this, image analysis was focused on the cell–cell junctions. The presence of the silica nanoparticles significantly altered the appearance of the cell–cell distances for all the conditions studied (± Pitstop 2, ± Mucus) as compared to controls, i.e., non-treated cells (Figures 4 and S8e for **D**_**35**_). Higher cell–cell distances were observed in the absence of Pitstop 2 and mucus (Figure 4b), with **NrNPs** being the material that induced the highest change in the cells’ separation. These data can be considered coherent with the notion that application of Pitstop 2 increased the rigidity of intestinal cells (Figure S4). Of note, **D**_**35**_ also increased cell–cell distances in comparison to the untreated control (Figure S8e). However, the measured values were much lower than those observed for **NrNPs** (Figure 4), although both nanostructures showed similar cell–particle interaction efficiencies. These results suggest that the larger aspect ratio of **NrNPs** had a greater influence on cell adjustment, which is in agreement with the systematic increase of the intercellular distances when the size of the spherical particles increased from **D**_**35**_ to **D**_**90**_ and **D**_**130**_.

**Figure 4:**
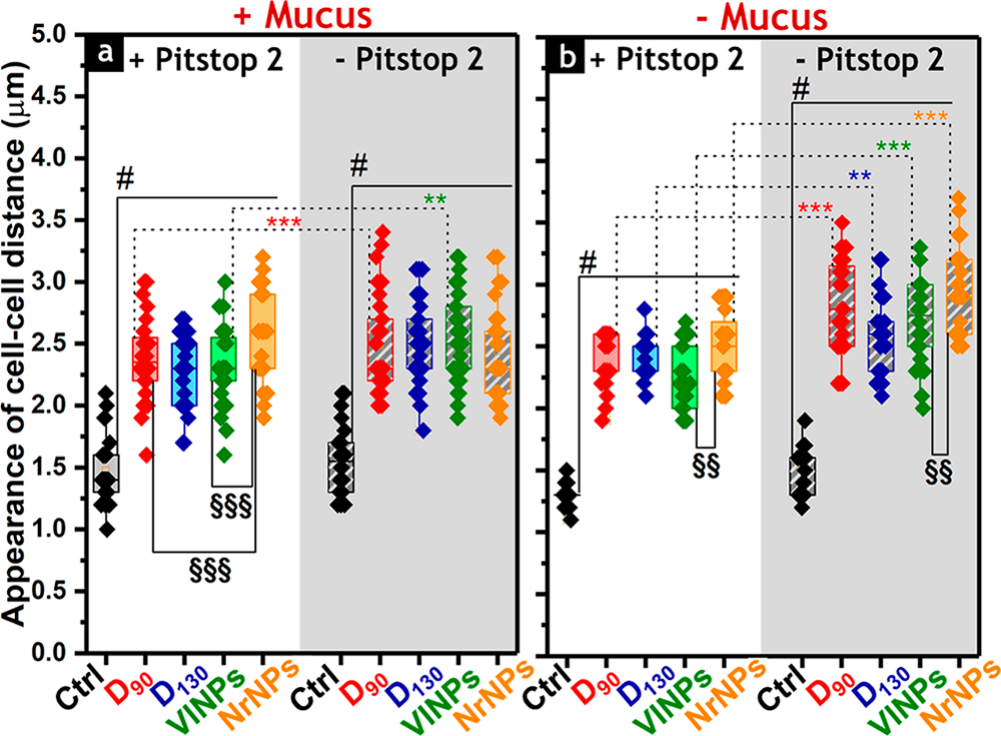
Appearance of cell–cell distances of Caco-2/HT29-MTX-E12 cells after 6 h of incubation with silica particles in (a) the presence and (b) the absence of the mucus layer, measured from *n* > 50 cells. Statistically significant differences according to one-way ANOVA and Fisher Test when treatments compare the same particle type (*) or different ones (§) are as follows: */§ (*p* < 0.5), **/§§ (*p* < 0.01), or ***/§§§ (*p* < 0.001). The appearance of cell–cell distances in controls (i.e., cells not treated with nanoparticles) was significantly different (#) from the values of all corresponding particle treatments (*p* < 0.001). All data were obtained from three independent cell preparations.

With the purpose of deepening our understanding of the mechanisms behind the interaction of shape-tailored materials with the intestinal cells, the behavior of **NrNPs** was evaluated in presence of selected molecules that are known to modify the architecture of the cell membrane (Figure 5). In this regard, using compounds that are potentially related to the diet, it was possible to model relevant interactions in the gastrointestinal compartment. First, the mycotoxin deoxynivalenol (DON) was used for cell treatment. DON is a food contaminant that inhibits protein biosynthesis^37^ and modifies membrane structures relevant for barrier function.^38^ Particularly for Caco-2 cells, application of DON increases the transepithelial electrical resistance and the expression of the junctional protein claudin-4, hence tightening the appearance of the cell monolayer.^39^ Second, cells were treated with methyl-β-cyclodextrin (mβCD), an inhibitor of the caveolae-mediated endocytosis as a complementary pathway to Pitstop 2 regulation. MβCD causes cholesterol depletion from the cell membrane, inducing the alteration of its organization and fluidity.^26,40^ Lastly, dietary fatty acid oleic acid (OA) was used. OA was previously used as modulator of the intestinal permeability to study drug delivery pathways.^41^ Additionally, OA alters membrane fluidity and rearranges the cytoskeleton of intestinal cells, thus modulating their mechanosensory apparatus.^42^

**Figure 5:**
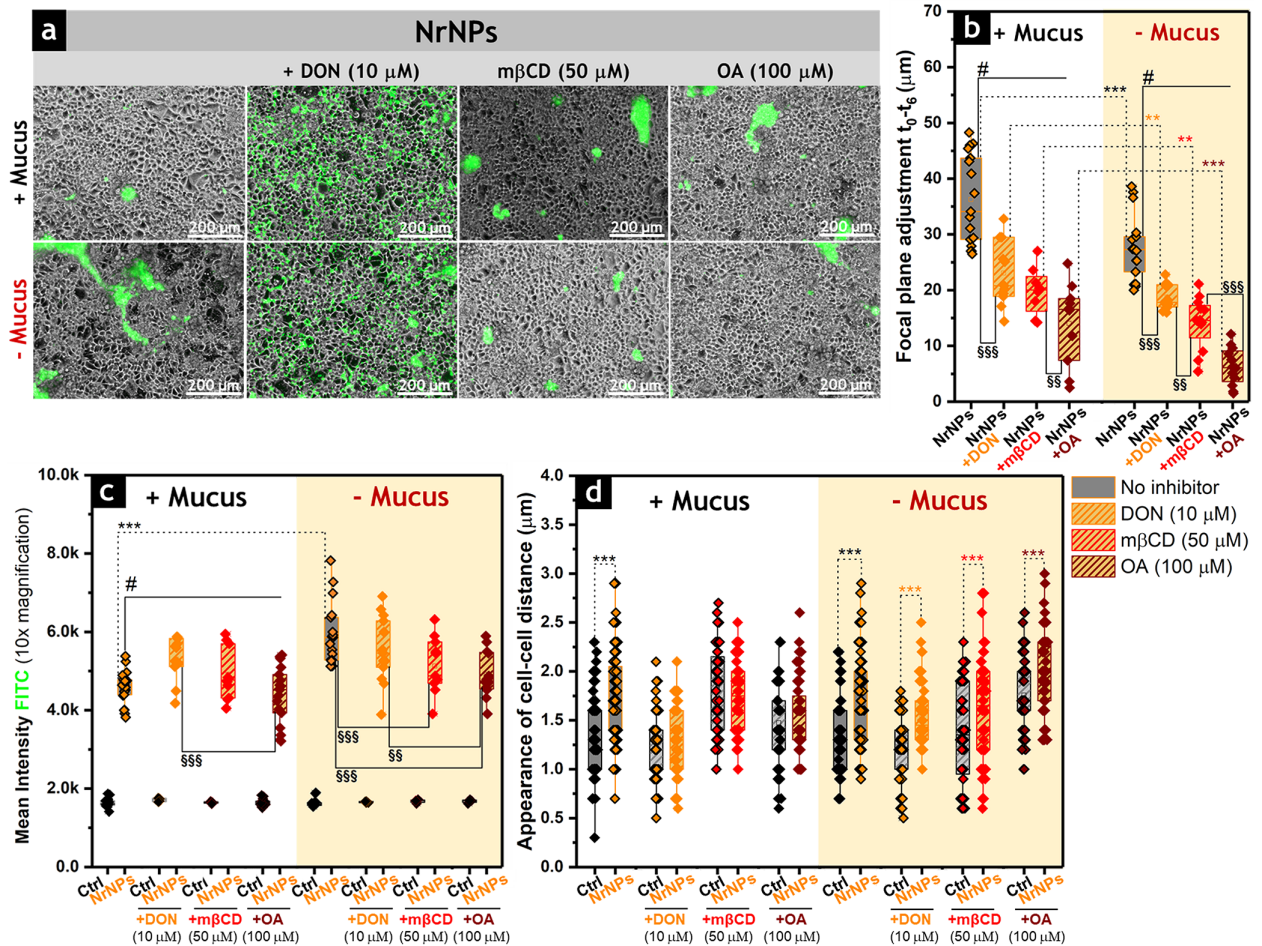
(a) Representative cell phase contrast fluorescence images (10× magnification) after cell treatment with FITC-labeled **NrNPs** in presence of DON (10 μM), mβCD (50 μM), and OA (100 μM). Scale bars represent 200 μm. (b) Quantification of the focal plane adjustment obtained from the difference between the optical parameters set immediately after FITC-labeled **NrNPs**-treatment (*t*_0_) and after 6 h of incubation (*t*_6_) for cells with or without the mucus layer in the presence of DON (10 μM), mβCD (50 μM), or OA (100 μM). Experiments were performed in biological triplicates, and at least 18 paired images were analyzed before and after focus adjustment (*n* = 18). All treatments were significantly different (#, p < 0.001) in comparison to controls (i.e., cells treated only with NrNPs). (c) Quantification of the mean fluorescence intensity of FITC (*n* = 18 optical fields) in the presence of DON (10 μM), mβCD (50 μM), or OA (100 μM). (d) Appearance of cell–cell distances of Caco-2/HT29-MTX-E12 cells (measured from *n* > 50 cells) after 6 h of incubation with **NrNPs** with and without mucus and in the presence of DON (10 μM), mβCD (50 μM), and OA (100 μM). Statistically significant differences according to one-way ANOVA and Fisher Test when particle treatments are compared in the same (*) or different conditions (§) are as follows: */§ (*p* < 0.5), **/§§ (*p* < 0.01), or ***/§§§ (*p* < 0.001). The mean fluorescence intensity of FITC in the controls (i.e., cells treated only with **NrNPs** but without DON, mβCD, or OA) was significantly different (#) from the values of all treatments (DON, mβCD, or OA, *p* < 0.001) with mucus. All data were obtained from three independent cell preparations.

The working concentrations were selected on the basis of literature data in order to induce changes in barrier function without affecting cell viability.^38,42,43^ After 20 h of treatment with DON, mβCD or OA, the cells were incubated for 6 h with the **NrNPs**. As depicted in Figure 5a, the cell monolayer preserved its integrity, and no cell detachment was observed. The positioning of the **NrNPs** was significantly affected in both the presence and absence of mucus (Figure 5b). The penetration of **NrNPs** through the mucus layer decreased from 34 μm in the negative controls (i.e., cells treated only with **NrNPs**) to 21, 20, and 17 μm after incubation with DON, mβCD, and OA, respectively. In absence of mucus, penetration depths of 18, 15, and 6 μm were measured for DON, mβCD, and OA treatments, respectively, in contrast to 27 μm obtained for the controls exposed only to **NrNPs** (Figure 5b). Additionally, in the absence of mucus, the chemical modulation of the membrane architecture was associated to a reduced mean fluorescence for **NrNPs** when applied to intestinal cells (mβCD and OA, Figure 5c). Cell treatment with OA affected more significantly both the penetration efficiency and interaction of **NrNPs** with the intestinal monolayer. In presence of mucus, intercellular distances increased after treatment with **NrNPs** with respect to the controls (i.e., non-treated cells, Figure 5d). This response was abolished in presence of DON, mβCD, and OA. With the removal of the mucus layer, **NrNPs** increased the cell–cell distances in all the experimental conditions. Extended comparison among different treatments can be found in Figure S9.

Since mβCD and OA returned the strongest modulation of the **NrNPs** behavior, these treatments were selected for proof of principle experiments with **D**_**130**_ and **VlNPs** (Figure S10, a and b). Despite the improved penetration efficiency of the **VlNPs** compared to **D**_**130**_ (Figure S10, c), the interaction with the cells was limited and lower mean fluorescence intensities were observed (Figure S10, d). This effect could be attributed to the adsorption of mucins and globular proteins that might cluster around the rough surface of the **VlNPs**, limiting their interaction with the cell membrane. This was corroborated from the increase of the hydrodynamic diameter of **VlNPs** in the cell culture medium (230 ± 1 nm) compared with **D**_**130**_ (180 ± 1 nm), despite the retention of high colloidal stability and low PDI values in the biological medium (Figure S7).

In summary, rod-shaped particles **NrNPs** diffused better through the mucus barrier and their larger aspect ratio had the greater influence on the paracellular permeation, possibly easing cell separation. Even though opening/adaption of the cell tight junctions can be assumed, this did not seem to be critical, since the integrity of the intestinal barrier was maintained with no toxicity or significant alteration of the transepithelial electrical resistance (TEER), as observed in Figure S11. Small particle size and surface roughness generally had a positive albeit more limited influence on diffusion through the mucus and interaction with the intestinal cells. The latter seemed to be strongly affected by mechanisms involving clathrin receptors, as underpinned by the results obtained with Pitstop 2. Despite not observing directly the cell-uptake-mediated mechanism, it was corroborated that these structures play a fundamental role in the adaptation of the cell membrane, modulating its rigidity and possibly biomechanical compliance. Complementary internalization routes could also modulate the interaction of silica with the cell monolayer, where the membrane integrity, modification of its architecture (tight junctions), organization, fluidity, and mechanosensory apparatus display a crucial role in the barrier maintenance. Overall, these data offer a precious insight into the complex relationship between the size and shape of nanomaterials and barrier function, which could strengthen biotechnological applications, including the development of more efficient therapeutic agents and novel alternatives for drug delivery through the intestinal epithelium.

## Supporting Information Available

Materials and methods; DLS profiles, colloidal stability, N_2_-physisorption isotherms, and NLDFT pore size distributions of **D**_**35**_, **D**_**90**_, **D**_**130**_, **VlNPs** and **NrNPs**; summary of the physicochemical parameters of the silica nanoparticles; live fluorescence images in presence of mucus; AFM maps and quantification of the cellular stiffness of the cell monolayer; phase contrast images acquired at 4× and 20× magnifications; colloidal stability of **D**_**35**_, **D**_**90**_, **D**_**130**_, **VlNPs** and **NrNPs** tested in cell culture medium containing mucus; TEM and SEM images of **D**_**35**_; fluorescence microscopy experiments; statistical comparison of the effect of treatments with DON, mβCD, OA, and **NrNPs** on the appearance of cell–cell distances; and TEER measurements, paracellular permeability of Lucifer Yellow, and Neutral Red assays for cell viability determination (PDF)
