# Supplementary material for: Morphology-Dependent Interaction of Silica Nanoparticles with Intestinal Cells: Connecting Shape to Barrier Function
Source: Nano Lett. 2023 Jul 11;23(16):7758–66. doi: 10.1021/acs.nanolett.3c00835 (PMC10450799; doi:10.1021/acs.nanolett.3c00835)
Supplement: Supplementary file 1 — nl3c00835_si_001.pdf [file nl3c00835_si_001.pdf]

Supporting Information

Morphology-dependent interaction of silica nanoparticles with intestinal cells: Connecting shape to barrier function

Claudia Iriarte-Mesa,[a,b] Maximilian Jobst,[b,c,d] Janice Bergen,[b,c,d] Endre Kiss,[c] Ryong Ryoo,[e] Jeong-Chul Kim,[f] Francesco Crudo,[d] Doris Marko,[d] Freddy Kleitz,[*a] Giorgia Del Favero[*c,d]

***Corresponding Authors: freddy.kleitz@univie.ac.at; giorgia.del.favero@univie.ac.at**

[a] *Department of Inorganic Chemistry – Functional Materials, Faculty of Chemistry, University of Vienna, Währinger Str. 42, 1090 Vienna, Austria*

[b] *Vienna Doctoral School in Chemistry (DoSChem), University of Vienna, Währinger Str. 42, 1090 Vienna, Austria*

[c] *Core Facility Multimodal Imaging, Faculty of Chemistry, University of Vienna, Währinger Str. 38-40, 1090 Vienna, Austria*

[d] *Department of Food Chemistry and Toxicology, Faculty of Chemistry, University of Vienna, Währinger Str. 38-40, 1090 Vienna, Austria*

[e] *Department of Energy Engineering, Korea Institute of Energy Technology (KENTECH), 21 KENTECH-gil, Naju 58330, Republic of Korea*

[f] *Center for Nanomaterials and Chemical Reactions, Institute for Basic Science (IBS), Daejeon 34141, Republic of Korea*

Table of contents

Materials 2

Methods 2

Synthesis of silica nanoparticles 2

Labelling of silica nanoparticles 3

Cell culture and seeding 4

Cell treatments 4

Fluorescence microscopy experiments 5

Atomic Force Microscopy (AFM) 6

Transepithelial Electrical Resistance (TEER) 6

Paracellular Permeability: Lucifer Yellow 7

Cell Viability: Neutral Red Assay 7

Characterization of the materials 8

Figures and Tables 9

Figure S1. DLS profiles and colloidal stability of **D_{35}**, **D_{90}**, **D_{130}**, **VlNPs** and **NrNPs** 9

Figure S2. N_2-physisorption isotherms and NLDFT pore size distributions (PSD)....9
Table S1. Physico-chemical parameters of the silica nanoparticles....10
Figure S3. Live cell fluorescence images of the interaction silica nanoparticles with intestinal cells....10
Figure S4. AFM maps and quantification of cellular stiffness of the cell monolayer....11
Figure S5. Live cell phase contrast fluorescence images (20x magnification) after cell treatment....12
Figure S6. Phase contrast images (4x magnification)....13
Figure S7. Colloidal stability of $\mathbf{D_{35}}$, $\mathbf{D_{90}}$, $\mathbf{D_{130}}$, **VlNPs** and **NrNPs** in cell culture medium....14
Figure S8. Interaction of $\mathbf{D_{35}}$ with intestinal cells in presence or absence of Pitstop 2....15
Figure S9. Statistical comparison of different **NrNPs**-treated cells with DON, mβCD and OA....16
Figure S10. Interactions of $\mathbf{D_{130}}$ and **VlNPs** with intestinal cells in presence of mβCD and OA....17
Figure S11. Cell permability and viability assays for intestinal cells treated with silica nanoparticles..18
References....19

Materials

Tetraethyl orthosilicate (TEOS, 98%), triethylamine (TEA, ≥99%), hexane (≥99%), cetyltrimethylammonium bromide (CTAB, 98%), cetyltrimethylammonium chloride (CTAC, 25 wt % H_2O), fluorescein isothiocyanate (FITC, ≥90%), and anhydrous toluene (99.8%) were purchased from Sigma Aldrich (Taufkirchen, Germany). Sodium hydroxide (NaOH, 1.0 N standardized solution), cyclohexane (ACS, ≥99%), ammonium hydroxide (NH_4OH, 28%), anhydrous ethanol (EtOH, 95%), *N*-acetyl-L-cysteine (BioReagent, suitable for cell culture), (3-aminopropyl)triethoxysilane (APTS, 98%) and dimethyl sulfoxide (DMSO, ≥99%) were purchased from Alfa Aesar (Massachusetts, USA). Sodium heptafluorobutyrate (FC_4) and hydrochloric acid (HCl, 37%) were purchased from VWR Chemicals (Pennsylvania, USA) and Fluorochem (Hadfield, UK), respectively. The cell-permeable clathrin inhibitor Pitstop 2 (ab120687) was purchased from Abcam Biochemicals (Cambridge, UK). Deoxynivalenol (DON) was purchased from Romer Labs (Tulln, Austria). Methyl-β-cyclodextrin (mβCD) was purchased from Sigma-Aldrich (St. Louis, US). Oleic acid (OA, O1383-1G) and Neutral Red dye were obtained from Sigma-Aldrich (Taufkirchen, Germany). Lucifer Yellow CH di-lithium salt was purchased from Santa Cruz Technologies (Dallas, TX, USA). Materials for cell culture and fluorescence imaging were purchased from GIBCO Invitrogen (Karlsruhe, Germany), Lonza Group Ltd (Basel, Switzerland), Sigma-Aldrich Chemie GmbH (Munich, Germany) and Sarstedt AG&Co (Nuembrecht, Germany).

Methods

Synthesis of silica nanoparticles. The synthesis of dendritic mesoporous silica nanoparticles (DMSNs) with different sizes was performed following the procedures described by Wang *et. al.*[1]

(**D_{35}** and **D_{90}**) and Juère *et. al.*[2] (**D_{130}**). First, TEA (68 mg), FC_4 (24.6 mg) and CTAB (380 mg) were dissolved in 25 mL of deionized water for the synthesis of **D_{35}**. The mixture was stirred (150 rpm) for 1 h at 25 °C. Instead, for the synthesis of **D_{90}**, half of FC_4 was used (49.2 mg) and the mixture with the same amounts of TEA, CTAB and water (as for **D_{35}** synthesis) was stirred (150 rpm) for 1 h at 80 °C. TEOS (4 mL) was then introduced drop wise, and the stirring (700 rpm) was kept for 24 h at 25 ºC (**D_{35}**), or for 2 h at 80 °C (**D_{90}**). The products (**D_{35}** and **D_{90}**) were isolated by centrifugation (9000 rpm, 20 min) and washed with ethanol three times. The particles were extracted for 2 h with 100 mL of EtOH and 1 drop of HCl (37%) at 90 °C. After drying overnight at 100 °C, **D_{35}** and **D_{90}** were calcined at 550°C for 5 h. For the synthesis of **D_{130}**, TEA (360 mg), CTAC (8 mL), and 72 mL of deionized water were mixed and stirred (150 rpm) for 1 h at 60 °C. Then, an organic mixture of hexane (32 mL) and TEOS (8 mL) was added drop wise, and the biphasic system was slowly stirred (150 rpm) at 60 °C, overnight. The organic phase was then removed, and the solution was centrifuged for 20 min at 10000 rpm. After drying at 100 °C overnight, the as-made **D_{130}** product was extracted for 2 h with 100 mL of EtOH and 1 drop of HCl (37%) at 90 °C. After drying overnight at 100 °C, the particles were calcined at 550°C for 5 h. The synthesis of virus-like silica particles (**VlNPs**) was performed following a procedure described by Wang *et. al.*[3] with slight modifications. NaOH (0.8 mL, 0.1 M) and CTAB (1 g) were dissolved in 50 mL of deionized water. The solution was stirred for 2 h at 60 °C, and 20 mL of a mixture of TEOS/cyclohexane (1:4 v/v) was introduced drop wise. The stirring (150 rpm) was kept for 7 days at 60 °C. The **VlNPs** were centrifuged and washed three times with ethanol, and the particles were extracted by washing three times with EtOH (50 mL containing 1 drop of HCl, 37%) and twice with water. After washing with absolute ethanol and drying overnight at 100 °C, the **VlNPs** were calcined at 550 °C for 5 h. The procedure reported by Yu *et. al.*[4] was followed for the synthesis of rod-like MSNs (**NrNPs**). CTAB (2.27 g) was dissolved in 280 mL of deionized water. NH_4OH (12 mL, 28%) was added and the solution was stirred for 1 h. TEOS (9.7 mL) was introduced at once, and the solution was then stirred (600 rpm) for 4 h at room temperature. The product (**NrNPs**) was centrifuged (9000 rpm, 20 min) and washed with ethanol three times. The surfactant template in the as-made **NrNPs** was removed following the same procedure described for **VlNPs** extraction. The **NrNPs** were dried overnight at 100 °C and calcined at 550 °C.

Labelling of silica nanoparticles. **D_{35}**, **D_{90}**, **D_{130}**, **VlNPs** and **NrNPs** were labeled with fluorescein isothiocyanate (FITC) based on a previously reported method.[5] First, a FITC-APTS silane was prepared by the covalent coupling of FITC with APTS. FITC (20 mg, 4 eq.) was dissolved in 10 mL of anhydrous EtOH, and APTS (3 µL, 1 eq.) was added at room temperature, under stirring (800 rpm) and argon atmosphere. The reaction was kept for 24 h in the dark. 100 mg of the silica nanoparticles (**D_{35}**, **D_{90}**, **D_{130}**, **VlNPs** or **NrNPs**) were degassed overnight at 150 °C and dispersed

in 30 mL of anhydrous toluene at 50 ºC under stirring (700 rpm). Subsequently, the FITC-APTS stock solution (50 µL) was added to each silica dispersion and the grafting was further carried out overnight. The FITC-labelled silica nanoparticles were recovered by centrifugation (9000 rpm, 20 min), washed once with toluene and three times with ethanol, and then dried at 35 °C for 24 h.

Cell culture. Caco-2 cells and HT29-MTX-E12 were purchased from ATCC and cultivated according to the specification of the supplier in DMEM (high glucose, 4.5 $g \cdot L^{-1}$) supplemented with fetal bovine serum (10 %), L-glutamine (1%, 2 mM), non-essential amino acids (1%) and penicillin-streptomycin (1%). The cells were incubated in a humidified incubator with 5% CO_2 at 37 °C. For the microscopy experiments, cells were seeded in Greiner Bio-One CELLview™ four compartment tissue culture dishes (growth area: 1.9 cm^2/compartment). Before the live cell imaging experiments, a total cell density of 85000 $cells \cdot cm^{-2}$ was seeded in each compartment using 500 µL of cell culture medium, which rendered approx. 161500 cells/compartment (145350 Caco-2 cells and 16150 HT29-MTX-E12 cells). The Caco-2/HT29-MTX-E12 co-culture was incubated at 37 °C for 7 days, replacing the cell culture medium with fresh medium every two days. At the end of the differentiation time, a homogenous layer of mucus was visible on the cell's monolayer.

Cell treatments. After 7-days incubation the cell medium was removed, and the cells were washed twice with DBPS. The clathrin inhibitor Pitstop 2 (8.25 mM in DMSO) was diluted in serum free medium (25 µM, 0.3% DMSO). This solution was added to the cells (500 µL/well), followed by 10 min-incubation at 37 °C according to the specification of the supplier (+ Pitstop 2). For the negative control (- Pitstop 2), 500 µL of serum free medium (including 0.3% DMSO) were used for the incubation. The cells were then washed twice with serum free medium, and 500 µL of freshly prepared dispersions of FITC-labelled nanoparticles, *i.e.*, $\mathbf{D_{35}}$, $\mathbf{D_{90}}$, $\mathbf{D_{130}}$, **VlNPs** and **NrNPs**, were added. The particle suspensions were previously prepared in serum free medium (320 $\mu g \cdot mL^{-1}$) according to a standard operating procedure (SOP). Suspensions were vortexed for 15 secs and placed in the ultrasonic bath for 20 min prior cell treatment to ensure stable and homogenous dispersion. The cells were incubated with the silica nanoparticles for 6 h at 37 °C. For the experiments including biochemical modulation of the cell junctions, (*i.e.*, DON, mβCD and OA treatments), cell medium was removed after 7 days-incubation, and the cells were washed twice with DPBS. Solutions of DON (10 µM, 0.5% DMSO), mβCD (50 µM, 0.05% DMSO) or OA (100 µM, 0.03% DMSO) were freshly prepared in serum free medium containing bovine serum albumin (BSA, 1 $mg \cdot mL^{-1}$) and added to the cell wells (500 µL/well), followed by 20 h-incubation at 37 °C. For the controls, 500 µL of serum free medium (containing BSA, 1 $mg \cdot mL^{-1}$) were used for the incubation. The cells were then washed twice with serum free medium (+ BSA, 1 $mg \cdot mL^{-1}$) and the treatment with FITC-labelled nanoparticles was applied following the

same procedure described above. For the experiments performed in absence of mucus, the procedure described by Behrens *et. al.*[6] was implemented for mucus removal. The cells were first washed with DBPS (550 µL/well) and *N*-acetyl-L-cysteine solution in serum free medium (10 mM, 500 µL/well) was added. After 1 h-incubation at 37 °C under agitation (220 rpm), the cells were washed twice with DPBS and treated according to the protocol described above for particle-cell interaction experiments in presence of the mucus layer and the inhibitor Pitstop 2, DON, mβCD or OA.

Fluorescence microscopy experiments. Live cell imaging experiments were performed on a confocal LSM microscope Zeiss 710 equipped with ELYRA PS.1 system and a Water Plan Apochromat 63x/1.2 objective. At the end of the treatments, the cells were washed twice with Live Cell Imaging Solution (LCI, 550 µL/well) to remove non-internalized FITC-labelled particles. Then, cells were incubated with CellMask™ Deep Red Plasma Membrane Stain (1:1000 dilution)[7] and DNA staining Hoechst 33342 (2 $\mu g \cdot mL^{-1}$).[8] After 15 min-incubation with the staining solution (250 µL/well) at 37 °C, the cells were washed twice with LCI (500 µL/well). After addition of LCI (400 µL/well), imaging was performed in Live Cell Imaging Solution (Molecular Probes, Life Technologies, Thermo Fisher Scientific, USA). The FITC-fluorescence signal was quantified from the maximum intensity projection of the respective channel in each 3D reconstruction obtained from z-stack imaging. For each condition (± Mucus; ± Pitstop 2) and particle treatment (*i.e.*, $\mathbf{D_{90}}$, $\mathbf{D_{130}}$, **VlNPs** and **NrNPs**), at least three images were analyzed (n=3) and obtained in three independent biological replicates.

For imaging experiments at lower resolution, 154700 cells (139230 Caco-2 cells and 15470 HT29-MTX-E12 cells) were seeded in 24 wells-cell culture plate (Sarstedt, 1.82 cm^2 growth area). Differentiation protocol was followed by mucus removal, pharmacological treatments (Pitstop 2, DON, mβCD and OA) and FITC-labelled particles treatments. Imaging of the cells treated immediately after the addition of the particles suspensions (t_0) and after 6 h-incubation (t_6) at 37 °C was performed with Lionheart FX Automated microscope from BioTek (Vermont, USA). Image acquisition and quantification was performed using GEN5 Microplate Reader and Imager Software Version 3.05 from BioTek (Vermont, USA). The images were composed from the phase contrast and GFP [469, 525 nm] channels. All experiments were carried out using at least 3 independent cell preparations (biological replicates) performed in technical duplicates. Quantification of the focal plane adjustment at 4x magnification was used to corroborate the observation that particles were able to penetrate through the mucus layer and was calculated from the difference between the focus value set immediately after FITC-labelled particles-treatment (t_0) and after 6 h-incubation (t_6). To confirm homogeneous particle distribution in the optical fields at the beginning of the experiments (t_0) and to evaluate the percentage of residual fluorescence detectable at the end of the incubation [(t_6)/(t_0)]%, images were compared immediately after the

cell treatment and before incubation at the same coordinates (4x magnification). At least 18 paired optical fields were obtained in three independent biological replicates. Mean fluorescence data were obtained quantifying the mean FITC-fluorescence intensity of at least 18 optical fields (n=18) acquired at 10x magnification. For the determination of cell-cell distances at least 16 phase contrast-fluorescence images (20x magnification) obtained in three independent biological replicates were analyzed (> 50 cells). Data groups were compared with the ANOVA test accepting as threshold values $p < 0.05$

Atomic Force Microscopy (AFM). For the AFM experiments 782000 cells (703800 Caco-2 cells and 78200 HT29-MTX-E12 cells) were seeded in tissue culture dishes 40 93040 (TPP Techno Plastic Products AG, Switzerland). The cells were incubated for 7 days in cell culture medium (2 mL/dish), followed by mucus removal and Pitstop 2 treatments. Measurements were done in pre-warmed (37 °C) live cell imaging solution (Invitrogen, USA, Ref: A14291DJ). Experiments were performed on the JPK NanoWizard 4 XP using PFQNM-LC-A-CAL tips (Bruker, Germany). The tips were precalibrated with spring constants ranging between 0.069 – 0.161 $N \cdot m^{-1}$. For cells without mucus QI mode acquisitions were optimized with the following settings: Setpoint: 0.15 nN; Z Length: 1000 nm; Z Speed: 100 $\mu m \cdot s^{-1}$; Pixel Time 25 ms; 128 x 128 pixels. Determination of the Young´s Modulus was performed with the JPK NanoWizard Data processing software. Image processing was performed on a squared region of interest (ROI) within the cell image. First, Baseline Subtraction was performed to remove the baseline offset in vertical deflection by calculating the average value of a defined part of the curve and subtracting it from the whole curve. This was followed by calculating the vertical tip position, by correcting the height signal accounting for the cantilever deflection, and giving the actual vertical tip position plotted against the force, instead of the piezo displacement. Reference Force Height was calculated to determine the height at 50 % of the applied set point force. Elasticity Fit was used to calculate the Young´s Modulus using the Hertz/Sneddon model[9,10] for a paraboloid tip shape (70 nm tip radius). The median of the resulting Young´s Moduli was calculated and used for further evaluations. Imaging of cells with intact mucus layer was hampered in QI™ mode. PeakForce Tapping® mode was privileged for this task with the following settings: Setpoint 0.1nN; IGain 150 Hz; PGain 0.0048; Frequency 250 Hz; Amplitude: 0.464 µm, Pixels 128x128, Line Rate 0.3 Hz. Data groups were compared with the Student´s t-test accepting as threshold values $p < 0.05$.

Transepithelial Electrical Resistance (TEER). TEER measurements were performed as previously described to assess the integrity of the cell monolayer.[11,12] To this aim, an Epithelial Voltohmmeter (EVOM2) coupled to a chopstick electrode pair (STX2, both World Precision Instruments, Sarasota, FL, USA) was used. Prior to measurements, the chopstick electrodes were rinsed with ethanol and equilibrated at room temperature in medium for 20 min.[12] TEER measurements were performed before and after the treatment with $\mathbf{D_{130}}$, **VlNPs** and **NrNPs** (320

µg·mL^{-1}) and subsequent incubation (37 ºC) for 6 h and 24 h. The mean value of three measurements was assessed per well and the TEER of the blank well was subtracted from the TEER of the treated wells. All samples values were presented as percentage of controls (*i.e.*, non-treated cells).

Paracellular Permeability: Lucifer Yellow. After 24 h-incubation with **D$_{130}$**, **VlNPs** and **NrNPs**, the small hydrophilic compound Lucifer Yellow was used to test the tightness of the monolayers and study changes in paracellular permeability of treated cells. For the assay, both compartments of the transwell system were washed with Hank's balanced salt solution (HBSS) buffer containing 25 mM D-glucose, 20 mM HEPES, 1.25 mM $CaCl_2$ and 0.5 mM $MgCl_2$ at pH 7.4. Subsequently, 0.5 mL of a 0.1 mg/mL Lucifer Yellow CH di-lithium salt solution in HBSS buffer and 1.5 mL of pure HBSS buffer were added to the apical and basolateral compartment, respectively. After a 1 h incubation at 37 °C, fluorescence of the basolateral medium of all wells as well as pure Lucifer Yellow solution were measured in triplicates (excitation: 485 nm, emission: 535 nm). The fluorescence of the sample wells was related to pure LY after subtraction of the blank (HBSS) and was reported as % permeability. In the solvent control, a Lucifer Yellow permeability < 1% was accepted for considering cell monolayers as tight.

Cell Viability: Neutral Red Assay. The Neutral Red Assay was performed as previously described.[13–15] Prior to cell treatment, 154700 cells (139230 Caco-2 cells and 15470 HT29-MTX-E12 cells) were seeded in 24 wells-cell culture plate (Sarstedt, 1.82 cm² growth area). The cells were incubated for 7 days in cell culture medium (500 µL/well). Thereafter, cells were incubated for 6 h with **D$_{35}$**, **D$_{90}$**, **D$_{130}$**, **VlNPs** and **NrNPs**, previously dispersed in complete cell culture medium (320 µg·mL^{-1}). For the measurement of the cell viability in absence of mucus, the mucus layer was previously removed according to the protocol described above. All experimental layouts included solvent and negative controls (*i.e.*, non-treated cells). Neutral Red dye was dissolved in Dulbecco's phosphate buffered saline (DPBS) to reach a stock concentration of 4 mg·mL^{-1}. The day before use, the Neutral Red stock solution was further diluted to 40 µg·mL^{-1} in cell culture medium (NR medium) and incubated at 37 ºC. To remove undissolved dye crystals, NR medium was centrifuged for 10 min at 600 × *g* and subsequently filtered with filter paper. After 6 h-incubation with the nanoparticles (*i.e.*, **D$_{35}$**, **D$_{90}$**, **D$_{130}$**, **VlNPs** and **NrNPs**), the cells were washed twice with DPBS (550 µL/well) and the medium was replaced by NR medium (500 µL/well), followed by 3 h incubation (37 ºC) before washing steps with DPBS (to remove unbound dye). Cells were then treated with 500 µL of de-staining solution (50:50:1 ethanol absolute, dH_2O, glacial acetic acid) and shaken for 10 min at 500 rpm on a plate shaker. After transferring 130 µL of de-staining solution to a fresh 96-well plate, absorbance was measured at 540 nm using a Cytation3 imaging reader (BioTek, Winooski, VT, USA). Results were related to the respective

solvent control (test/control [%]) data groups were compared with the Student´s t-test accepting as threshold values $p < 0.05$.

Characterization of the materials. Transmission electron microscopy (TEM) images were recorded with a Titan G2 ETEM (FEI) at an accelerating voltage of 300 kV. The samples for TEM imaging were prepared by dropping EtOH containing the suspended powder sample (4 µL) on a holey carbon film-coated 300 mesh copper grid. Scanning electron microscopy (SEM) images were taken using a Verios 460 field emission scanning electron microscope (FEI) at an accelerating voltage of 5 kV and a decelerating voltage of 4 kV to have a landing voltage of 1 kV. The samples for SEM imaging were dispersed on a carbon tape and kept under vacuum for 1 h before imaging. Dynamic light scattering (DLS) analyses and zeta-potential measurements were performed on a Malvern DTS Nano Zetasizer at a 173° scattering angle (equilibrium time set at 3 min, 3 measurements for each sample). To guarantee correct calibration before zeta-potential measurements, a standard suspension (carboxylate modified polystyrene latex microspheres) with a zeta-potential of −40 (± 6) mV was measured. The samples were dispersed in H_2O with a concentration of 0.7 mg·mL^{-1}, vortex (10 min) and sonicated (90 min) prior to the analysis. For the measurements of colloidal stability of **D_{35}**, **D_{90}**, **D_{130}**, **VlNPs** and **NrNPs** in the presence of mucus, the particles were dispersed in the cell culture medium isolated after 7 days-incubation of Caco-2/HT29-MTX-E12 cells (320 µg·mL^{-1}). N_2-physisorption isotherms were measured at -196 °C (77 K) using an Autosorb-iQ3 sorption analyzer (Anton Paar, Boynton Beach, USA). Prior to the analysis, calcined **D_{35}**, **D_{90}**, **D_{130}**, **VlNPs** and **NrNPs** were outgassed 10 h at 150 °C. The specific surface area (S_{BET}) was determined using the Brunauer-Emmet-Teller (BET) equation in the relative pressure range 0.05 - 0.2. The total pore volume was determined at $P/P_0 = 0.95$. The pore size distributions were calculated using the non-local density functional theory (NLDFT) method on the (metastable) adsorption branch, considering an amorphous SiO_2 (oxide) surface and a cylindrical pore model. The calculations were carried out using ASiQwin 5.2 software provided by Anton Paar Quantatech Inc.

Figures and Tables

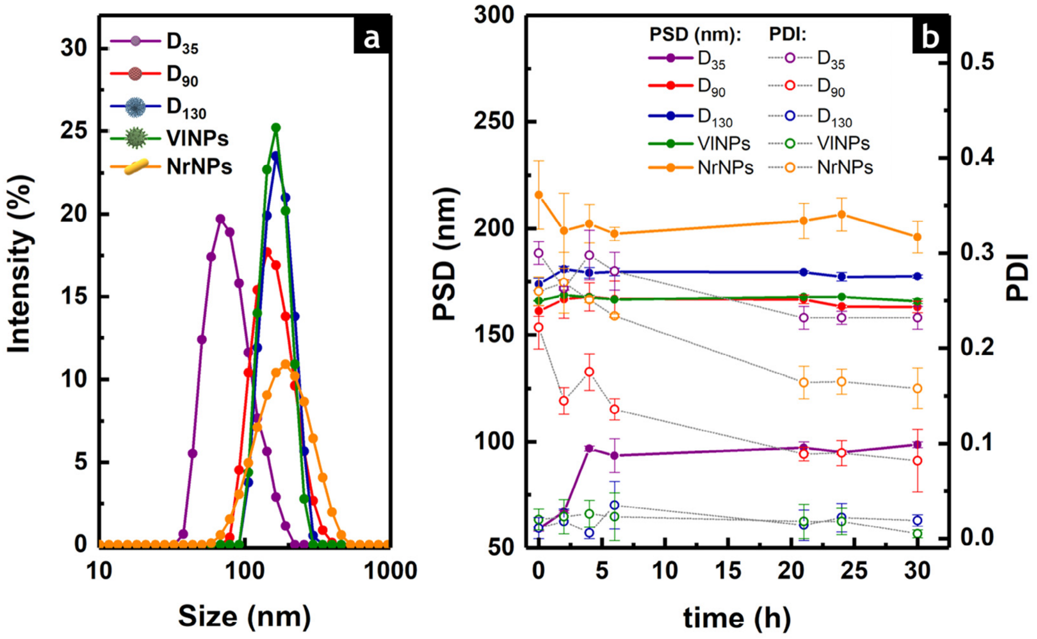

Figure S1. (a) DLS profiles of the silica nanoparticles, *i.e.*, **D$_{35}$**, **D$_{90}$**, **D$_{130}$**, **VINPs** and **NrNPs**, dispersed in H_2O (0.7 mg·mL^{-1}) (b) Colloidal stability tested *via* DLS analysis. All the silica materials exhibited a narrow particle size distribution (PSD), stable over time, in correspondence with the consistently low polydispersity index (PDI), below 0.3.

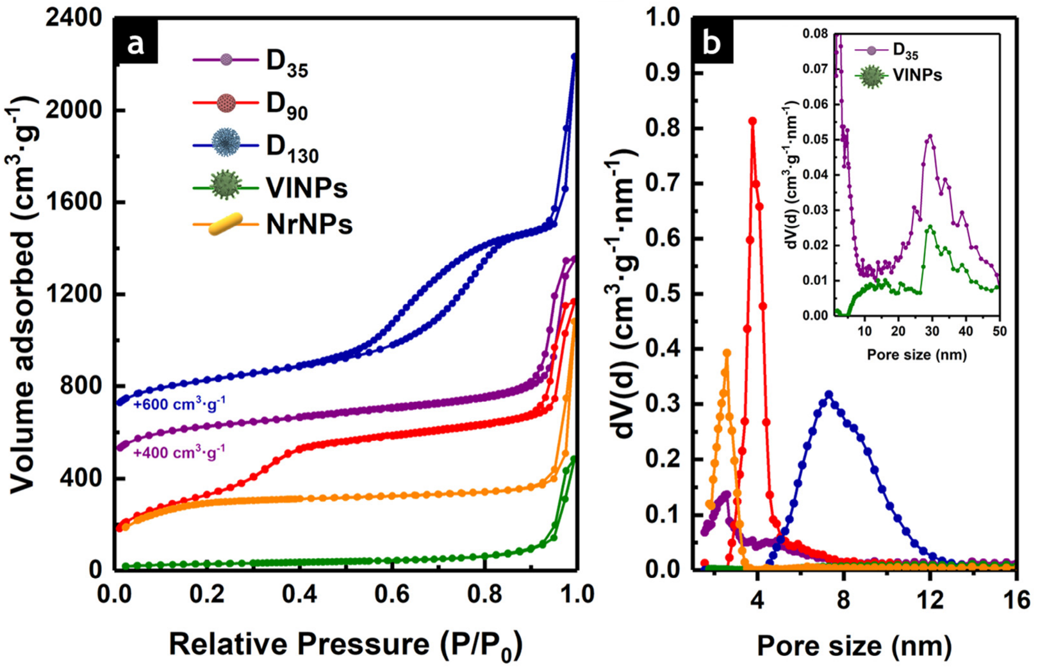

Figure S2. (a) N_2-physisorption isotherms measured at – 196 °C (77 K) of **D$_{35}$** (purple), **D$_{90}$** (red), **D$_{130}$** (blue), **VINPs** (green) and **NrNPs** (orange). (b) Respective NLDFT pore size distributions. *Inset:* Pore size distributions of **D$_{35}$** and **VINPs** exhibiting a broad peak centered at 29 nm.

Table S1. Physico-chemical parameters of the different silica nanoparticles.

Material	S_{BET} [a] ($m^2 \cdot g^{-1}$)	Pore size [a] (nm)	Pore volume[a] ($cm^3 \cdot g^{-1}$)	Particle size[b] (nm)	Zeta-potential (mV)
**D_{35}**	807	2.6<br>29 (broad)	1.39	55 (± 1)	-28.1 (± 0.4)
**D_{90}**	1223	3.8	1.65	161 (± 2)	-41.5 (± 0.8)
**D_{130}**	806	7.3	2.03	174 (± 3)	-50.1 (± 0.5)
**VINPs**	95	29 (broad) [c]	0.61	166 (± 1)	-39.8 (± 0.7)
**NrNPs**	1101	2.6	1.17	216 (± 3)	-49.0 (± 0.5)

[a] Porosity data (*i.e.*, specific surface area S_{BET}, pore volume and pore size) were obtained from the N_2-physisorption analysis (77 K). [b] The particle size was measured using DLS. [c] **VINPs** did not exhibit a mesoporous ordering, as previously reported.[16] The porosity stems from interparticle voids and contributions from spaces between the spikes. The pore size distribution is broad and centered in 29 nm, as observed in Figure S2b, *inset*.

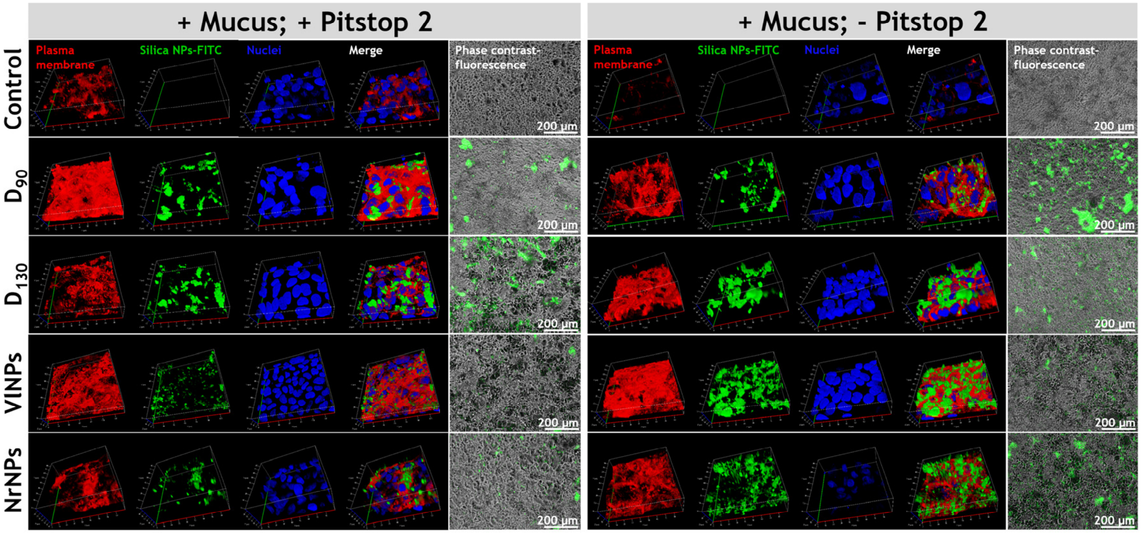

Figure S3. Representative live cell fluorescence images of the interaction of FITC-labelled silica nanoparticles with Caco-2/HT29-MTX-E12 cells after 6 h-incubation in presence of mucus, and previous treatment with or without Pitstop 2. In the 3D reconstructions (63x magnification) the scale bar segmentation is 10 µm and the plasma membrane is represented in red, the fluorescence coming from FITC in green, and the nuclei in blue. In the phase contrast images (10x magnification) scale bars stand for 200 µm.

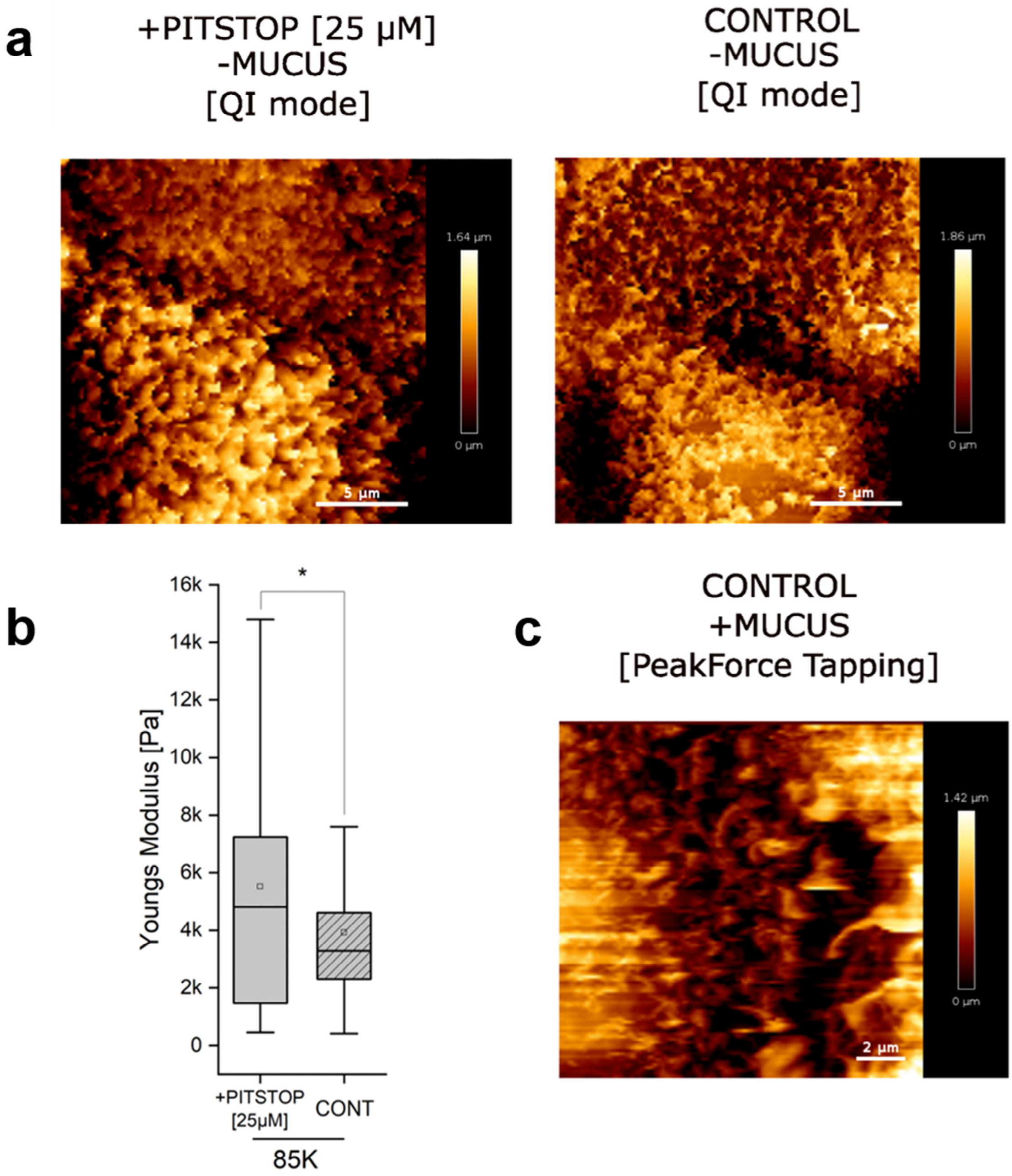

Figure S4. Atomic force microscopy maps and quantification of cellular stiffness of the Caco-2/HT29-MTX-E12 monolayer seeded at the same density as for the imaging experiments (85K). (a) Representative height maps taken in QI™ mode of the cell monolayer after mucus removal, after Pitstop 2 treatment (25 µM) and the correspondent control. At least n = 69 individual cells were evaluated, taken from at least 3 independent cell preparations (biological replicates) per condition. (Scale bar = 5 nm, color scale indicates the relative height in µm). (b) Quantification of the Young´s Modulus (YM; [Pa]). Results are given as the median YM per cell. Statistical significance is given as Student´s t-test (*$p < 0.05$). (c) Representative force maps taken using PeakForce Tapping® mode with intact mucus layer. (Scale bar = 2µm, color scale indicates the relative height in µm).

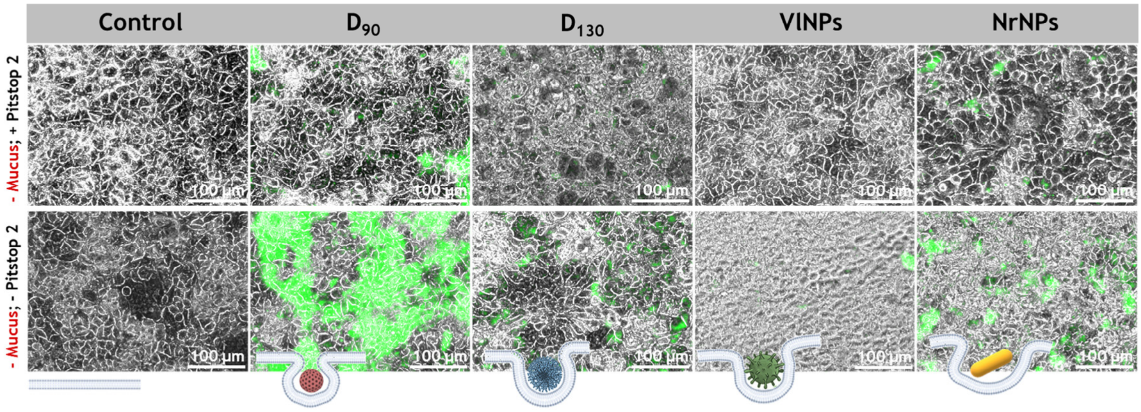

Figure S5. (a) Appearance of the Caco-2/HT29-MTX-E12 cells after treatment with FITC-labelled particles in absence of mucus (20x magnification). Scale bars stand for 100 µm.

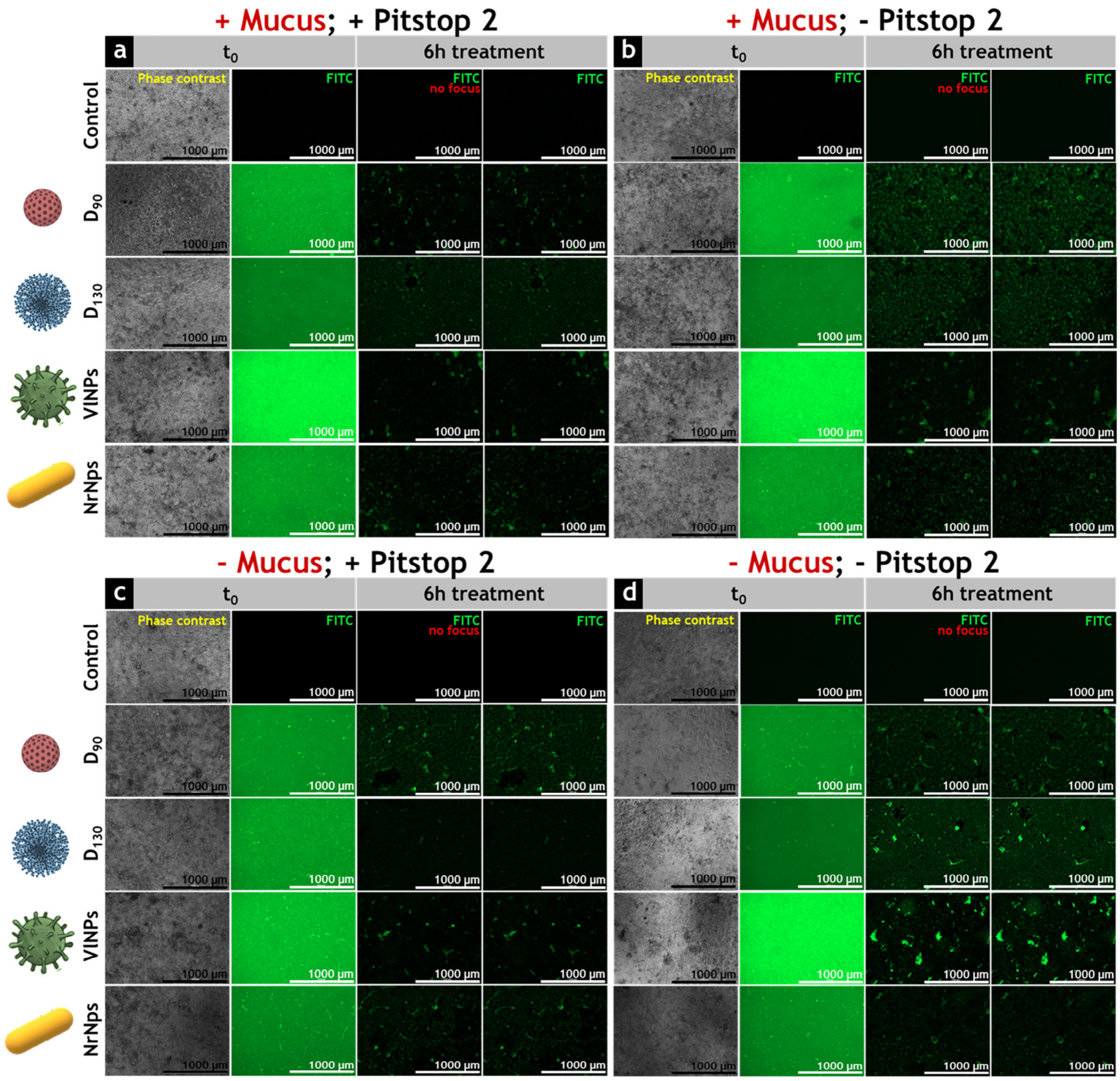

Figure S6. Phase contrast images (4x magnification) obtained immediately after the silica nanoparticles-treatment (t_0) and after 6 h-incubation at 37 ºC (t_6). The images were acquired at the same coordinates in both time-points of the assay, and the change in the focus position correspondent to the FITC-labelled particles (GFP [469, 525 nm] channel) was detected after 6 h-incubation. The particle-cell interactions were tested under different conditions: (a) + Mucus, + Pitstop 2; (b) + Mucus, - Pitstop 2; (c) - Mucus, + Pitstop 2; and (d) - Mucus, - Pitstop 2. Scale bars stand for 1000 µm.

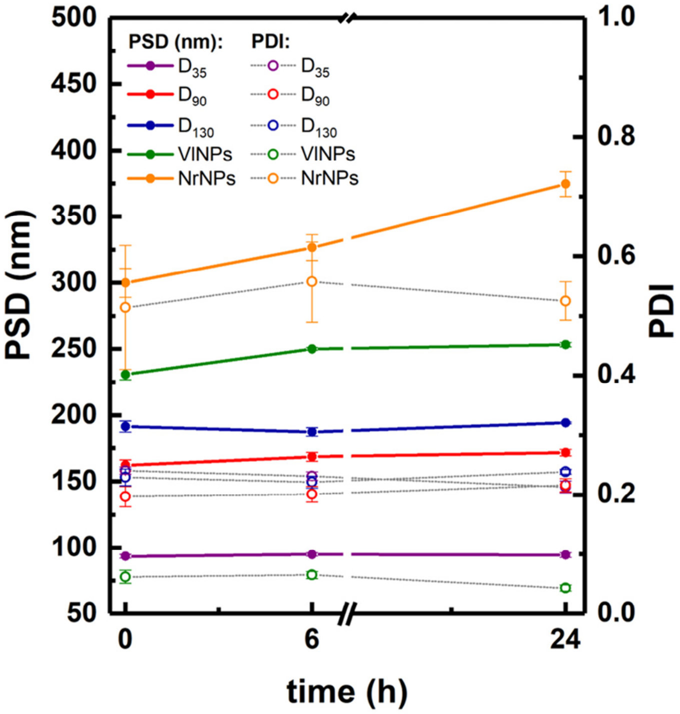

Figure S7. Colloidal stability tested *via* DLS analysis. The experiments were performed in cell culture medium containing mucus (silica particle concentration*:* 320 µg·mL^{-1}). Spherical and virus-like particles exhibited a narrow particle size distribution (PSD), stable over time, in correspondence with the consistently low polydispersity index (PDI), below 0.3. The higher PDI values obtained for **NrNPs** (above 0.3) indicate lower colloidal stability in biological medium.

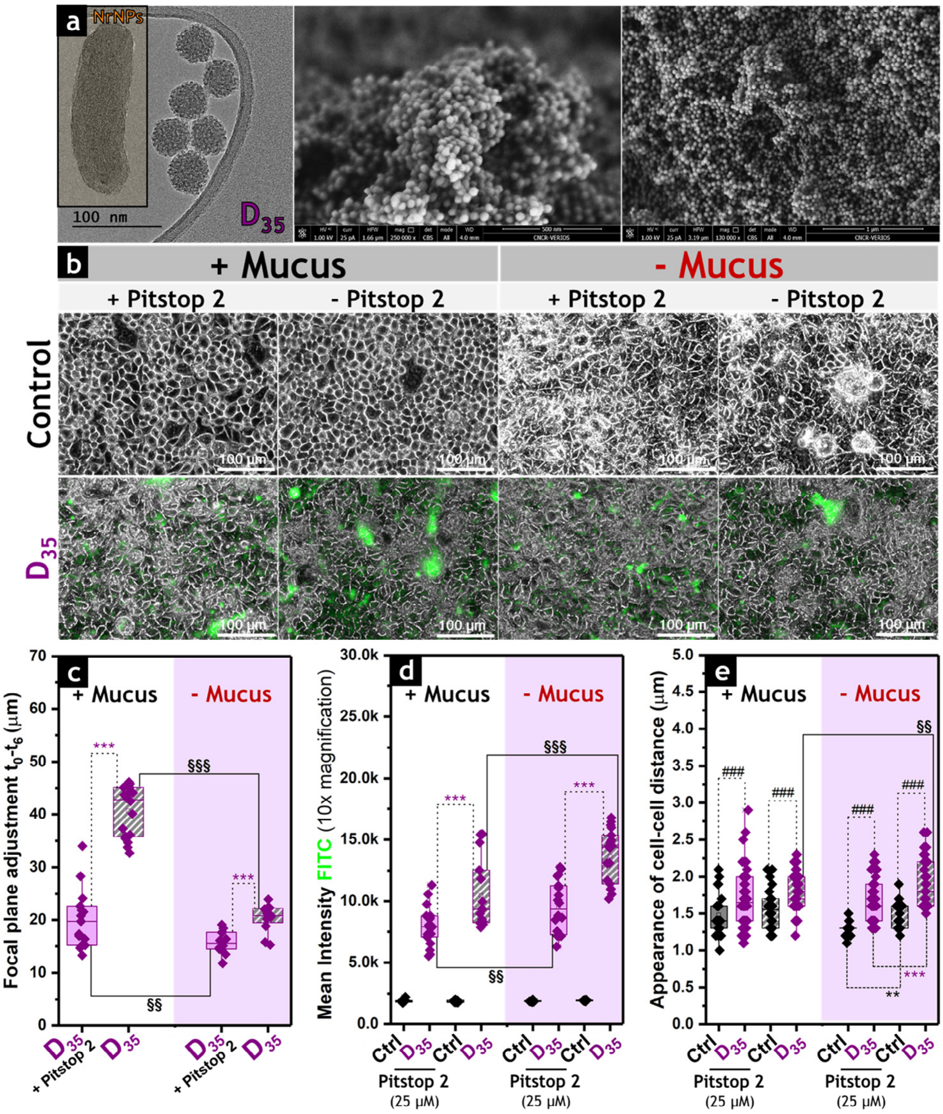

Figure S8. (a) Representative transmission electron microscopy (TEM) and scanning electron microscopy (SEM) images of **D35** obtained at different magnifications. Scale bars stand for 100 nm, 500 nm and 1 µm, respectively. *Inset:* TEM of **NrNPs**, where the matching of the thickness of the rod-like particles with the **D35** diameter is observed. (b) Representative cell phase contrast fluorescence images (20x magnification) after cell treatment with FITC-labelled **D35**. Scale bars stand for 100 µm (c) Quantification of the focal plane adjustment obtained from the difference between the optical parameters set immediately after FITC-labelled **D35**-treatment (t_0) and after 6 h-incubation (t_6) of cells with or without mucus layer. Experiments were performed in technical and biological replicates and at least 18 paired images were analyzed before and after focus adjustment (n = 18). (d) Quantification of the mean fluorescence intensity of FITC from phase-contrast fluorescence in presence and absence of mucus (n = 18 optical fields). (e) Appearance of cell-cell distances of Caco-2/HT29-MTX-E12 cells after 6 h-incubation with **D35** in presence or absence of the mucus layer (measured from n > 50 cells). Statistically significant differences according to One-way ANOVA and Fisher Test when the effect of Pitstop 2 (*) or mucus (§) is compared for different treatments, are represented by */§ (p <0.5), **/§§ (p <0.01), or ***/§§§ (p < 0.001). The appearance of cell-cell distances in controls (*i.e.*, non-treated cells) was significantly different (###) from the values of particle-treatments in all the conditions tested (p < 0.001).

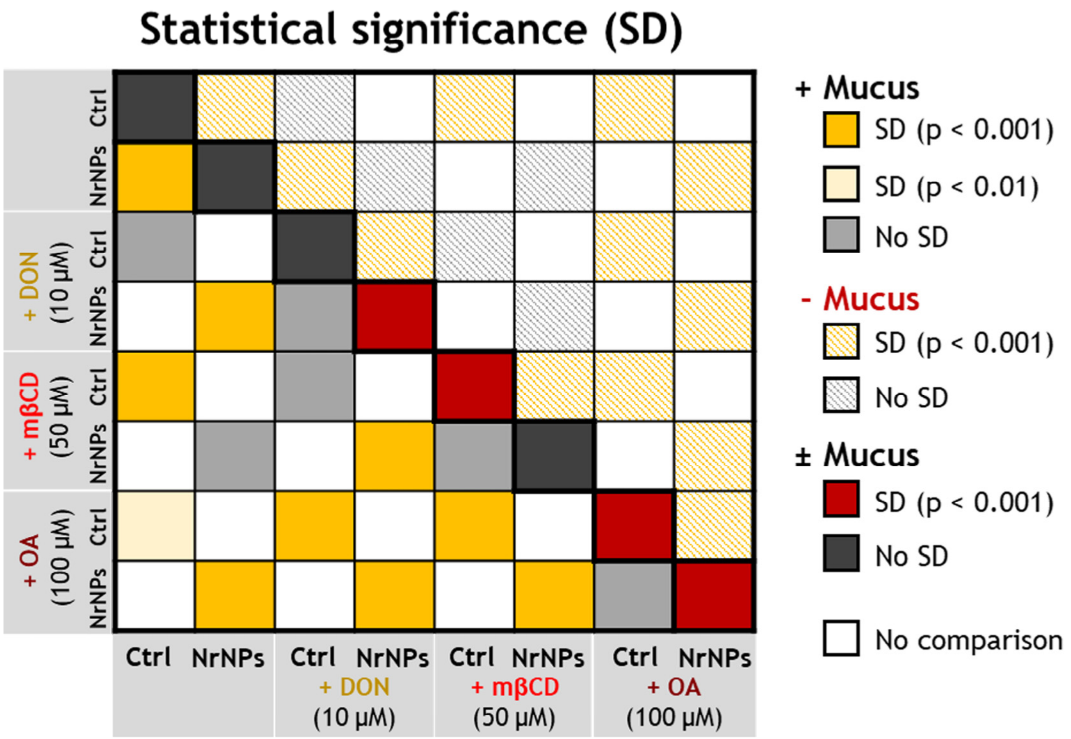

Figure S9. Extended statistical comparisons among the treatment groups. Significant differences according to One-way ANOVA and Fisher Test of appearance of cell-cell distance after cell-treatment with **NrNPs** in the presence of DON (10 µM), mβCD (50 µM) and OA (100 µM). Controls (*i.e.*, non-treated cells) and **NrNPs**-treatments (with or without DON, mβCD and OA) were compared both in presence (full colored squares) or absence of mucus (dashed colored squares). Comparison among the treatments (*i.e.*, DON, mβCD or OA with or without **NrNPs**) in presence or absence of mucus (± Mucus) is depicted in the diagonal.

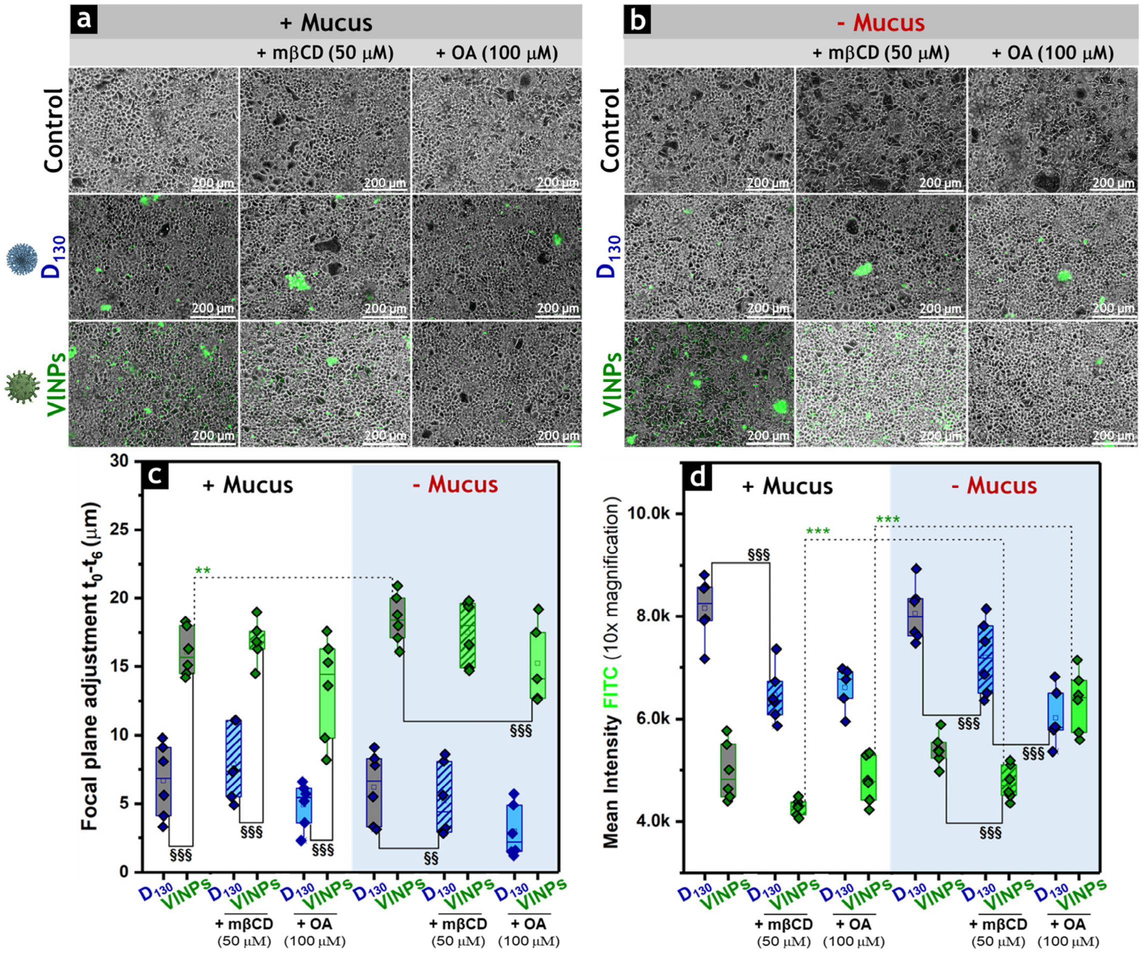

Figure S10. Representative cell phase contrast fluorescence images (10x magnification) after cell treatment with FITC-labelled **D_{130}** or **VINPs** in presence of mβCD (50 μM) and OA (100 μM) with (a) and without (b) mucus. Scale bars stand for 200 μm. (c) Quantification of the focal plane adjustment obtained from the difference between the optical parameters set immediately after FITC-labelled particle-treatment (t_0) and after 6 h-incubation (t_6) of cells with or without mucus layer in the presence of mβCD (50 μM) and OA (100 μM). Experiments were performed in three biological replicates and at least 18 paired images were analyzed before and after focus adjustment (n = 18). (d) Quantification of the mean fluorescence intensity of FITC from phase-contrast fluorescence with and without mucus (n = 18 optical fields) and in the presence of mβCD (50 μM) and OA (100 μM). Statistically significant differences according to One-way ANOVA and Fisher Test when the treatments compare the effect of mucus (*) or different particle-morphologies/cell junction's modulators (§), are represented by */§ ($p < 0.5$), **/§§ ($p < 0.01$), or ***/§§§ ($p < 0.001$).

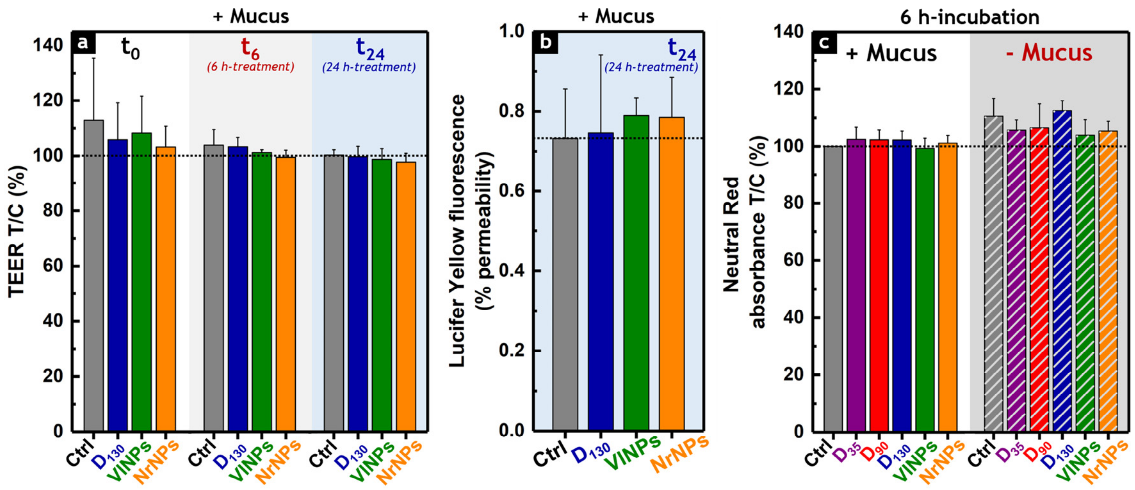

Figure S11. (a) Transepithelial electrical resistance (TEER) of Caco-2/HT29-MTX-cells before and after 6 h and 24 h of incubation with **D_{130}**, **VlNps**, **NrNPs** or cell culture medium (control, *i.e.,* non-treated cells). Results were normalized to the control (dashed line). (b) Lucifer Yellow fluorescence intensity in the basolateral compartment after incubation of Caco-2/HT29-MTX-E12 cells for 24 h with **D_{130}**, **VlNps**, **NrNPs** or cell culture medium (control, *i.e.,* non-treated cells). (c) Cell viability of Caco-2/HT29-MTX-E12 cells measured by Neutral Red assay after 6 h of incubation with silica nanoparticles in the presence or absence of mucus. Results are presented as means + standard deviations, normalized to the control (*i.e.*, non-treated cells), and measured in 3 biological replicates performed in technical duplicate. Student's *t* test did not show significant differences for the permeability or cytotoxicity assays ($p > 0.05$ for all the compared treatments).

References

(1) Wang, Y.; Song, H.; Yu, M.; Xu, C.; Liu, Y.; Tang, J.; Yang, Y.; Yu, C. Room Temperature Synthesis of Dendritic Mesoporous Silica Nanoparticles with Small Sizes and Enhanced MRNA Delivery Performance. *J. Mater. Chem. B* **2018**, *6* (24), 4089–4095. https://doi.org/10.1039/c8tb00544c.

(2) Juère, E.; Caillard, R.; Marko, D.; Del Favero, G.; Kleitz, F. Smart Protein-Based Formulation of Dendritic Mesoporous Silica Nanoparticles: Toward Oral Delivery of Insulin. *Chem. - A Eur. J.* **2020**, *26* (23), 5195–5199. https://doi.org/10.1002/chem.202000773.

(3) Wang, W.; Wang, P.; Tang, X.; Elzatahry, A. A.; Wang, S.; Al-Dahyan, D.; Zhao, M.; Yao, C.; Hung, C. Te; Zhu, X.; Zhao, T.; Li, X.; Zhang, F.; Zhao, D. Facile Synthesis of Uniform Virus-like Mesoporous Silica Nanoparticles for Enhanced Cellular Internalization. *ACS Cent. Sci.* **2017**, *3* (8), 839–846. https://doi.org/10.1021/acscentsci.7b00257.

(4) Yu, M.; Wang, J.; Yang, Y.; Zhu, C.; Su, Q.; Guo, S.; Sun, J.; Gan, Y.; Shi, X.; Gao, H. Rotation-Facilitated Rapid Transport of Nanorods in Mucosal Tissues. *Nano Lett.* **2016**, *16* (11), 7176–7182. https://doi.org/10.1021/acs.nanolett.6b03515.

(5) Hohagen, M.; Guggenberger, P.; Kiss, E.; Kählig, H.; Marko, D.; Del Favero, G.; Kleitz, F. TANNylation of Mesoporous Silica Nanoparticles and Bioactivity Profiling in Intestinal Cells. *J. Colloid Interface Sci.* **2022**, *623*, 962–973. https://doi.org/10.1016/j.jcis.2022.05.035.

(6) Behrens, I.; Vila Pena, A. I.; Alonso, M. J.; Kissel, T. Comparative Uptake Studies of Bioadhesive and Non-Bioadhesive Nanoparticles in Human Intestinal Cell Lines and Rats: The Effect of Mucus on Particle Adsorption and Transport. *Pharm. Res.* **2002**, *19* (8), 1185–1193. https://doi.org/10.1023/A:1019854327540.

(7) Del Favero, G.; Woel, L.; Janker, L.; Neuditschko, B.; Seriani, S.; Gallina, P.; Sbaizero, O.; Gerner, C.; Marko, D. Deoxynivalenol Induces Structural Alterations in Epidermoid Carcinoma Cells A431 and Impairs the Response to Biomechanical Stimulation. *Sci. Rep.* **2018**, *8*, 11351–11368. https://doi.org/10.1038/s41598-018-29728-5.

(8) Engelberg, S.; Modrejewski, J.; Walter, J. G.; Livney, Y. D.; Assaraf, Y. G. Cancer Cell-Selective, Clathrin-Mediated Endocytosis of Aptamer- Decorated Nanoparticles. *Oncotarget* **2018**, *9* (30), 20993–21006. https://doi.org/10.18632/oncotarget.24772.

(9) Sneddon, I. N. The Relation between Load and Penetration in the Axisymmetric Boussinesq Problem for a Punch of Arbitrary Profile. *Int. J. Eng. Sci.* **1965**, *3* (1), 47–57. https://doi.org/10.1016/0020-7225(65)90019-4.

(10) Reine, J. Über Die Berührung Fester Elastischer Körper (On the Contact of Elastic Solids). *Angew Math* **1882**, *92*, 156–171. https://doi.org/10.1515/crll.1882.92.156.

(11) Groestlinger, J.; Seidl, C.; Varga, E.; Del Favero, G.; Marko, D. Combinatory Exposure to Urolithin A, Alternariol, and Deoxynivalenol Affects Colon Cancer Metabolism and Epithelial Barrier Integrity in Vitro. *Front. Nutr.* **2022**, *9*, 882222. https://doi.org/10.3389/fnut.2022.882222.

(12) Beisl, J.; Varga, E.; Braun, D.; Warth, B.; Ehling-Schulz, M.; Del Favero, G.; Marko, D. Assessing Mixture Effects of Cereulide and Deoxynivalenol on Intestinal Barrier Integrity and Uptake in Differentiated Human Caco-2 Cells. *Toxins (Basel).* **2021**, *13* (3), 189. https://doi.org/10.3390/TOXINS13030189.

(13) Beisl, J.; Pahlke, G.; Abeln, H.; Ehling-Schulz, M.; Del Favero, G.; Varga, E.; Warth, B.; Sulyok, M.; Abia, W.; Ezekiel, C. N.; Marko, D. Combinatory Effects of Cereulide and Deoxynivalenol on in Vitro Cell Viability and Inflammation of Human Caco-2 Cells. *Arch. Toxicol.* **2020**, *94* (3), 833–844. https://doi.org/10.1007/s00204-020-02658-w.

(14) Beisl, J.; Pahlke, G.; Ehling-Schulz, M.; Del Favero, G.; Marko, D. Cereulide and Deoxynivalenol Increase LC3 Protein Levels in HepG2 Liver Cells. *Toxins (Basel).* **2022**, *14* (2), 1–16. https://doi.org/10.3390/toxins14020151.

(15) Repetto, G.; del Peso, A.; Zurita, J. L. Neutral Red Uptake Assay for the Estimation of Cell Viability/ Cytotoxicity. *Nat. Protoc.* **2008**, *3* (7), 1125–1131. https://doi.org/10.1038/nprot.2008.75.

(16) von Baeckmann, C.; Rubio, G. M. D. M.; Kählig, H.; Kurzbach, D.; Reithofer, M. R.; Kleitz, F. Evaporation-Induced Self-Assembly of Small Peptide-Conjugated Silica Nanoparticles. *Angew. Chem. Int. Ed.* **2021**, *60*, 22882–22887. https://doi.org/10.1002/ange.202108378.
